# Pharmacophore Modeling and 3D-QSAR Study of Indole and Isatin Derivatives as Antiamyloidogenic Agents Targeting Alzheimer’s Disease †

**DOI:** 10.3390/molecules25235773

**Published:** 2020-12-07

**Authors:** Rosa Purgatorio, Nicola Gambacorta, Marco Catto, Modesto de Candia, Leonardo Pisani, Alba Espargaró, Raimon Sabaté, Saverio Cellamare, Orazio Nicolotti, Cosimo D. Altomare

**Affiliations:** 1 Department of Pharmacy-Pharmaceutical Sciences, University of Bari Aldo Moro, Via E. Orabona 4, 70125 Bari, Italy; rosa.purgatorio@uniba.it (R.P.); nicola.gambacorta1@uniba.it (N.G.); modesto.decandia@uniba.it (M.d.C.); leonardo.pisani@uniba.it (L.P.); saverio.cellamare@uniba.it (S.C.); orazio.nicolotti@uniba.it (O.N.); cosimodamiano.altomare@uniba.it (C.D.A.); 2 Institute of Nanoscience and Nanotechnology (IN2UB), Department of Physical Chemistry, Faculty of Pharmacy, University of Barcelona, Joan XXIII 27-31, E-08028 Barcelona, Spain; aespargaro@ub.edu (A.E.); raimon.sabate@gmail.com (R.S.)

**Keywords:** isatin hydrazones, indole derivatives, antiamyloid agents, multitarget-directed ligands, 3D-QSAR

## Abstract

Thirty-six novel indole-containing compounds, mainly 3-(2-phenylhydrazono) isatins and structurally related 1*H*-indole-3-carbaldehyde derivatives, were synthesized and assayed as inhibitors of beta amyloid (Aβ) aggregation, a hallmark of pathophysiology of Alzheimer’s disease. The newly synthesized molecules spanned their IC_50_ values from sub- to two-digit micromolar range, bearing further information into structure-activity relationships. Some of the new compounds showed interesting multitarget activity, by inhibiting monoamine oxidases A and B. A cell-based assay in tau overexpressing bacterial cells disclosed a promising additional activity of some derivatives against tau aggregation. The accumulated data of either about ninety published and thirty-six newly synthesized molecules were used to generate a pharmacophore hypothesis of antiamyloidogenic activity exerted in a wide range of potencies, satisfactorily discriminating the ‘active’ compounds from the ‘inactive’ (poorly active) ones. An atom-based 3D-QSAR model was also derived for about 80% of ‘active’ compounds, i.e., those achieving finite IC_50_ values lower than 100 μM. The 3D-QSAR model (encompassing 4 PLS factors), featuring acceptable predictive statistics either in the training set (*n* = 45, q^2^ = 0.596) and in the external test set (*n* = 14, r^2^_ext_ = 0.695), usefully complemented the pharmacophore model by identifying the physicochemical features mainly correlated with the Aβ anti-aggregating potency of the indole and isatin derivatives studied herein.

## 1. Introduction

Alzheimer’s disease (AD) represents a major cause of illness and death in the world, especially in developed countries. Epidemiological data for United States account for almost 6 million Americans (aged ≥ 65) currently hit by AD and 122,000 deaths in 2018 [1]. Most recent data for European Union, collected in the 2019 Yearbook of Alzheimer Europe organization, report about 9 million patients in 2018, expected to increase to 10 and 16 million in 2025 and 2050, respectively, being Italy the country with the highest prevalence in population (>2%) [2].

Unfortunately, current therapeutic approaches for AD remain still limited to the symptomatic treatment of memory impairment by acetylcholinesterase (AChE) inhibitors, namely donepezil, rivastigmine and galantamine, and of glutamate excitotoxicity by memantine. The lack of disease-resolving therapies for AD still fuels the research in this field, despite poor results so far obtained in clinical trials that have discouraged pharmaceutical industries to pursuing the research for new drugs. There are about 120 trials currently registered in US, almost half of which are from drug repurposing, and most of which addressing disease-modifying targets and/or immunotherapy [3]. On the other hand, alternative hypotheses for the treatment of AD, considering the amyloid aggregation and toxicity, are still pursued mainly in academic settings. Despite many pitfalls registered in preclinical trials [4], there is still high interest in small molecules able to disrupt one or more steps in the amyloid cascade [5]. They may be resumed into a unitary approach known as amyloid hypothesis in AD, encompassing beta amyloid (Aβ) formation from APP precursor [6], Aβ aggregation in the amyloid cascade from toxic oligomers to senile plaques [7], tau protein aggregation [8], and tau hyperphosphorylation [9] as potential therapeutic targets. Considering such a number of pathophysiological events, the so-called multitarget approach, aiming at hitting two or more of AD-related targets with a unique chemical entity, has become the “new paradigm” of researchers in the field [10].

In this context, our research programs have long been focused on multitarget approach having the antiamyloidogenic activity as the central feature [11,12,13,14]. Particularly, since 2010 we investigated the chemical space around the isatin/indolinone molecular core [15,16,17], disclosing potent in vitro inhibitors of beta amyloid aggregation, acting at different stages of the fibrillogenesis [18,19]. Herein, we further proceeded with a ligand-based approach to design novel inhibitors, and evaluated the antiamyloidogenic activity of such new compounds, as well as their potential as multitarget agents for AD. In parallel, we exploited the data of published compounds from our previous screening campaigns to generate a reliable pharmacophore, complemented with a 3D-QSAR model, for Aβ aggregation inhibition by indole/indolone (mainly isatin hydrazones) derivatives.

**Figure 1:**
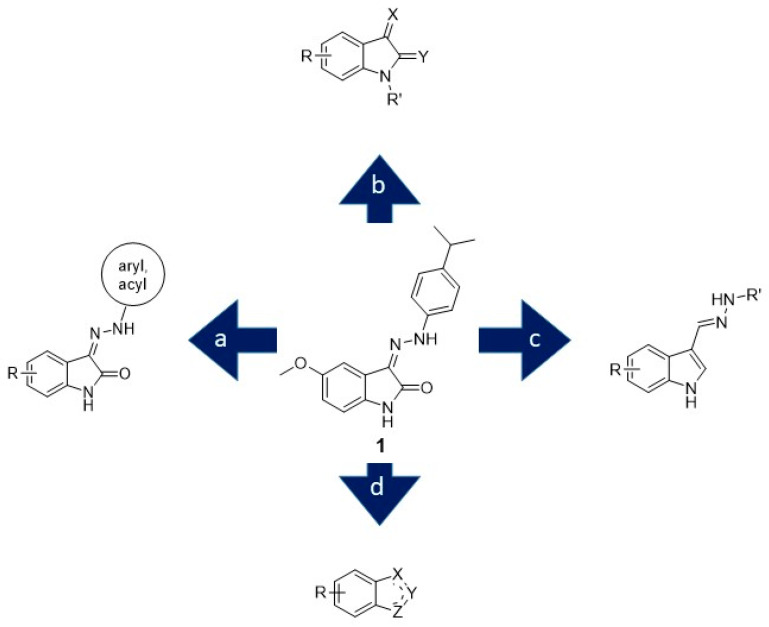
Ligand-based design of new inhibitors of amyloid aggregation.

Starting from the hit compound **1** [16,17] (Figure 1), the design strategy followed four main synthetic approaches aimed at (a) extending the exploration of the isatin-3-arylhydrazone scaffold; (b) simplifying the molecular structure to smaller isatin derivatives, possibly bearing *N*-substitution; (c) exploring bioisosteric indole derivatives, namely indole-3-carbaldehyde hydrazones; (d) investigating new indoline- and phthalimide-based analogues.

## 2. Results and Discussion

### 2.1. Chemistry

**Scheme 1:**
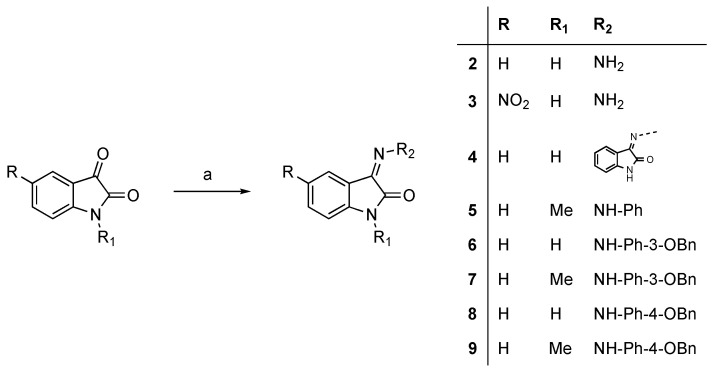
Synthesis of hydrazones **2**–**9**. *Reagents and conditions*: (a) NH_2_NH_2_•H_2_O, MeOH, reflux or ArNHNH_2_, MeOH, room temperature.

**Scheme 2:**
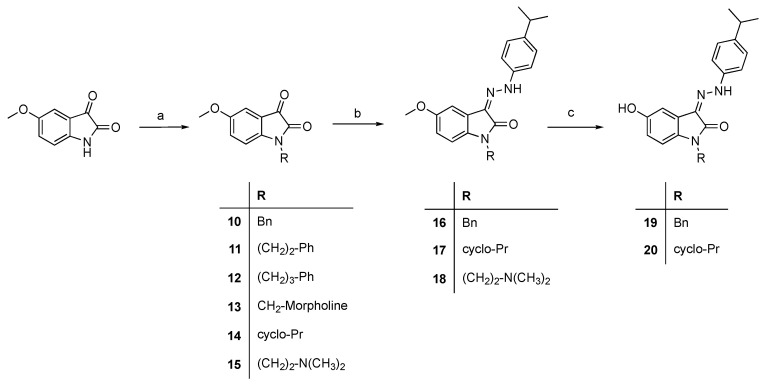
Synthesis of compounds **10**–**20**. *Reagents and conditions*: (a) DMF, K_2_CO_3_, 2-chloro-*N*,*N*-dimethylethylamine hydrochloride, rt, or formaldehyde, morpholine, ethanol, reflux; (b) MeOH, 4-isopropylphenylhydrazine, rt; (c) BBr_3_, CH_2_Cl_2_, −70 °C.

Hydrazones **2**–**4** were prepared by condensation of suitable isatin with hydrazine hydrate in refluxing methanol, whereas arylhydrazones **5**–**9** were synthesized from isatin or *N*-methylisatin with the corresponding phenylhydrazines at room temperature (Scheme 1). The *N*-alkylation of 5-methoxyisatin was carried out through different methods giving the derivatives **10**–**15**. The condensation of **10**, **14** and **15** with 4-isopropylphenylhydrazine afforded compounds **16**–**18**. Subsequently, **16** and **17** were demethylated with BBr_3_ [16] to **19** and **20**, respectively (Scheme 2).

**Scheme 3:**
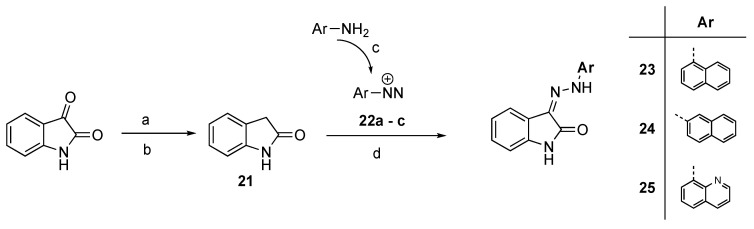
Synthesis of **23**–**25**. *Reagents and conditions*: (a) NH_2_NH_2_•H_2_O, EtOH; (b) NaOH, EtOH, H_2_O, reflux; (c) NaNO_2_, H_2_O, HCl 6N, 0 °C; (d) MeOH, CH_3_COONa, 0 °C.

**Scheme 4:**
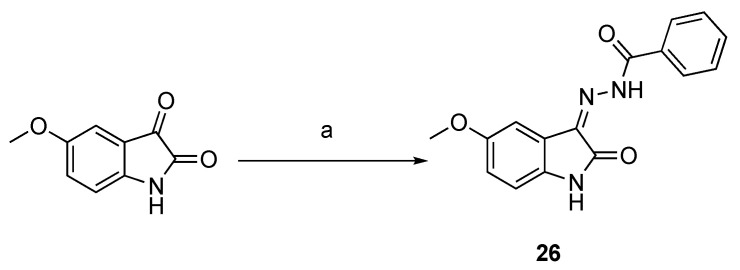
Synthesis of compound **26**. *Reagents and conditions*: (a) benzhydrazide, methanol, rt.

**Scheme 5:**
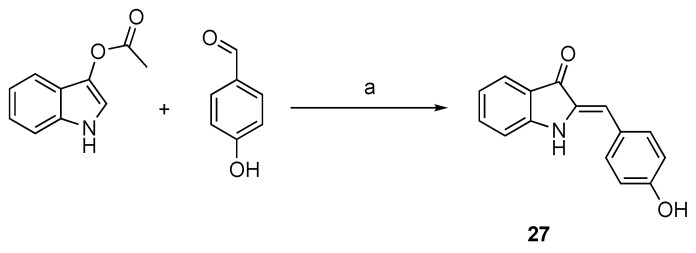
Synthesis of **27**. *Reagents and conditions*: (a) MeOH, HCl conc., reflux, N_2_.

The azo-coupling of aryldiazonium salts **22a**–**c** with indolin-2-one **21** [20] resulted in the formation of arylhydrazones **23**–**25** (Scheme 3). Compound **26** was synthesized by the condensation of 5-methoxyisatin with benzhydrazide as previously described [19] (Scheme 4), while the indolin-3-one **27** was obtained from indoxyl acetate and 4-hydroxybenzaldehyde in acidic conditions (Scheme 5).

**Scheme 6:**
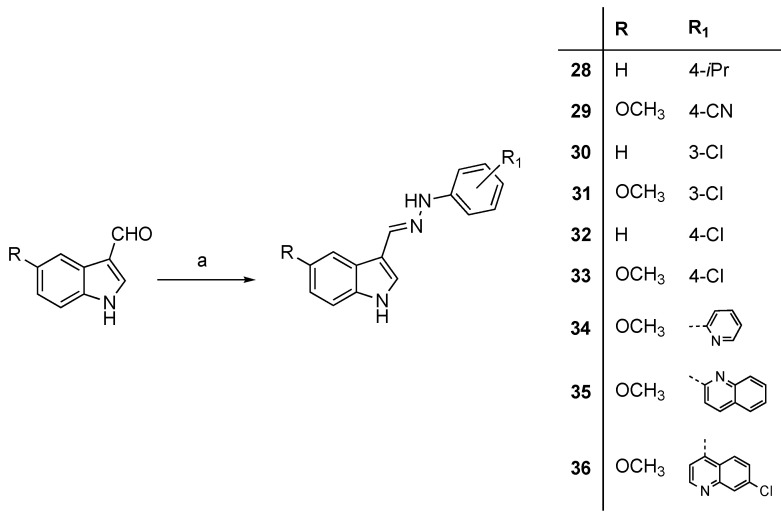
Synthesis of compounds **28**–**36**. *Reagents and conditions*: (a) arylhydrazine HCl, MeOH or EtOH, rt.

**Scheme 7:**
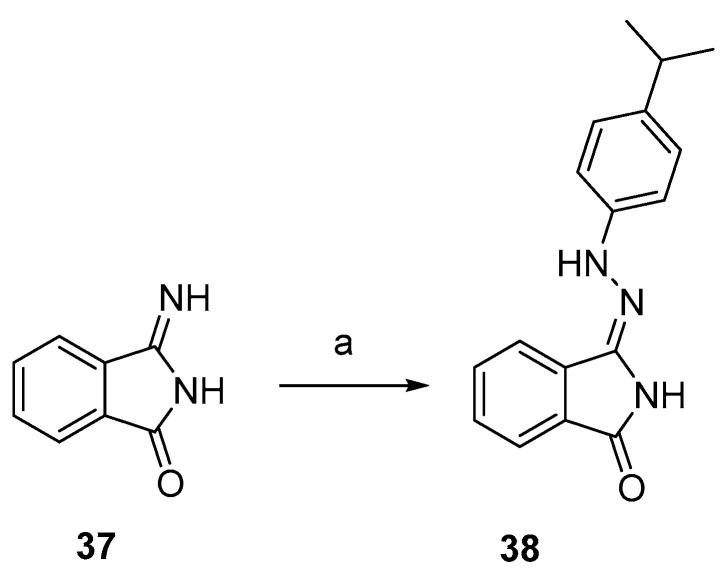
Synthesis of compound **38**. *Reagents and conditions*: (a) 4-isopropylphenylhydrazine, MeOH, rt.

The synthesis of indole-3-carbaldehyde hydrazones **28**–**36** was accomplished, according to previously reported procedures [19], by condensation of the R-substituted indole-3-carbaldehyde with arylhydrazine at room temperature (Scheme 6). The reaction of **37** with 4-isopropylphenylhydrazine, in the same conditions, afforded phthalimide monohydrazone **38** (Scheme 7).

**Scheme 8:**
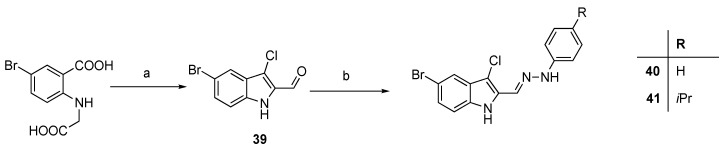
Synthesis of **40** and **41**. *Reagents and conditions*: (a) DMF, POCl_3_; (b) R-phenylhydrazine, acetic acid, EtOH, 0 °C.

**Scheme 9:**
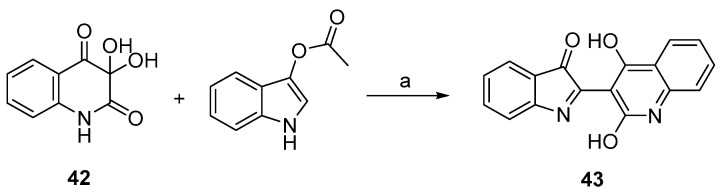
Synthesis of **43**. *Reagents and conditions*: (a) neat, 120 °C.

Compounds **40** and **41** were synthesized from **39**, prepared under Vilsmeier conditions [21], with the appropriate phenylhydrazine in an acid-catalyzed reaction (Scheme 8). Finally, the synthesis of the indolinone **43** was carried out under neat conditions starting from indoxyl acetate and dihydroxyquinolinedione **42** [22] (Scheme 9). Compounds **44** [23] and **45** [24] were prepared according to the quoted references.

### 2.2. Inhibition of Aβ_40_ Aggregation

All the newly prepared compounds were tested in a routine in vitro assay of inhibition of Aβ_40_ self-aggregation [25]. For the sake of clarity, compounds and related data are listed in four tables, according to the design strategy above mentioned. Table 1 collects all the compounds prepared with the aim of extending the exploration of SAR around the arylhydrazone substituent. The most potent compounds were found among the *N*-substituted analogues of **1**, namely **18** and **19**, while *N*-cyclopropyl derivative **20** resulted poorly active. Naphthylhydrazones **23** and **24** were also found highly active, while the quinoline derivative **25**, isoster of **23**, resulted less potent and the benzhydrazide **26** almost inactive. The 5-substitution appeared to have contrasting effects on potency; it is worthy of note that 5-methoxy analogue of 5-hydroxy derivative **20**, previously published [19], returned very higher potency than the latter, with IC_50_ in the submicromolar range.

The molecular simplification around the isatin core led to compounds listed in Table 2. In all cases, activities were quite far from reference compound **1**, particularly for unsubstituted hydrazones **2** and **3**. Also in this series, *N*-substituted derivatives of isatin scored the best results, with potencies increasing from benzyl to phenethyl and 3-phenylpropyl (**10** < **11**, **12**). Disappointingly, the morpholine derivative **13**, prepared as the Mannich base of 5-methoxyisatin, proved to be a weak inhibitor of aggregation, as well as the cyclopropyl derivative **14**. The substitution at the position 2 of isatin, leading to condensation products **27** and **44**, aimed at probing steric effects in this position. Both compounds retained only a weak activity against Aβ aggregation.

A major objective of this work was to investigate the isosteric variation of hydrazone derivatization from isatin to indole, afforded by condensing indole-3-carbaldehyde with selected arylhydrazines. The Aβ aggregation data in Table 3 account for a satisfactory activity profile, partially associated to the favorable effects of 5-methoxy substituent, and likely depending upon the hydrophobic feature of the arylhydrazine moiety. Both isopropyl and chlorine substituents warranted good potencies compared to nitrile, the 4-chloro isomer **32** being the most potent of the molecular subset. As a confirmation of the favorable hydrophobic effects, quinoline derivatives **35** and **36** were much more potent than pyridine congener **34**. As already noticed for compound **26**, benzhydrazide **45** lost any antiaggregating activity.

Finally, a few isomers of isatin were explored in order to ascertain the effects of modification of the core structure, while maintaining the arylhydrazone moiety (Table 4). Only compound **40** gave a measurable IC_50_ equal to 25 μM, while 4-isopropyl derivatives **38** and **41** were found less potent and the condensation product **43** was almost inactive.

### 2.3. Cytoprotection from Tau Toxicity

The deposition of aggregates of hyperphosphorylated tau protein constitutes the intracellular neurofibrillary tangles, a major histological evidence of AD. The role of tau hyperphosphorylation and aggregation in AD has established both of them as a target for therapeutic intervention [8], although only *N,N,N′,N′*-tetramethyl-10*H*-phenothiazine-3,7-diaminium (TRX0237, leucomethyl-thioninium), the reduced form of methylene blue dye, acting as an inhibitor of tau aggregation, has reached the clinical phase III [26]. In recent years, some of us developed an efficient cell-based assay of tau aggregation, a simple and inexpensive method that provides a straightforward assay to evaluate the putative anti-aggregation capacity of small molecules [27,28,29]. This method relies on the use of bacteria overexpressing amyloid-prone proteins to monitor the amyloid aggregation, thus constituting a physiologically relevant model. In particular, it exploits amyloid properties of the inclusions bodies (IBs) formed by tau full-length protein in bacteria stained by thioflavin S (ThS), a specific amyloid dye, to address the bacterial amyloid aggregation and the effect of anti-amyloid compounds in this polymerization process.

From a randomized screening of our in-house library, including four amyloid aggregation inhibitors **S22**, **S23**, **S34** and **S35** coming from a previous work [16], isatin hydrazones **S23** and **S35** emerged as interesting inhibitors of tau cytotoxicity, with IC_50_ values in the micromolar range (Table 5). We then decided to prepare and test, as analogues of **S23**, the bis-isatin hydrazone **4** and the 5-unsubstituted derivative **5**, along with the 5-unsubstituted congeners of **S35** and **S34**, i.e., compounds **6** and **8**. To confirm the effects of *N*-substitution at the isatin nitrogen, we also prepared and tested their *N*-methyl derivatives **7** and **9**. From the screening, isatin hydrazone **5** emerged as a potent inhibitor of tau aggregation (IC_50_ 4.6 μM), with slightly higher potency than its 5-methoxy congener **S23**. The remaining new compounds scored low inhibition values, despite the limited structural variations performed. On the other hand, the assay on Aβ_40_ aggregation disclosed the strong inhibitory potency of *N*-methyl derivatives **7** and **9**, with IC_50_s in the low to submicromolar range. Compared with their reference analogues **S35** and **S34**, where the activities of *meta* were slightly higher than *para* congeners, **6** and **8** showed an opposite trend, and both resulted strongly weaker than their corresponding *N*-methylated derivatives. Overall, compound **9** emerged as the most potent inhibitor of Aβ_40_ aggregation within the whole series of tested herein.

### 2.4. Evaluation of Multitarget Activity

The benefits of multitarget approach for the treatment of neurodegenerative diseases are widely recognized, although the pharmacological proof of concept is still controversial [10]. Our research has devoted large attention to dual inhibitors of cholinesterases (ChEs) and monoamine oxidases (MAOs), two major targets in neurodegeneration [30,31,32]. Acetyl-(AChE) and butyrylcholinesterase (BChE) are responsible for the hydrolysis of neurotransmitter acetylcholine, particularly in brain regions involved in learning and memory processes. AChE inhibitors still remain the first options for the symptomatic treatment of cognitive impairment in AD. Monoamine oxidase isoforms A (MAO A) and B (MAO B) are mitochondrial enzymes responsible for oxidative degradation of amine neurotransmitters and xenobiotics. Selective MAO A inhibitors are second-line drugs in the treatment of depression and mood disorders, while MAO B-selective inhibitors are used in the therapy of Parkinson’s disease and have neuroprotective effects. Isatin is an endogenous biofactor, metabolically derived from indole, largely present in the CNS [33]; isatin derivatives are recognized bioactive molecules for many neurological diseases [34]. 5-Substituted isatins have been described as potent MAO inhibitors [35,36] and recently, *N*-substituted 3-acylhydrazones of isatin showed interesting dual AChE-MAO B inhibitory activities [37].

The above-cited evidences prompted us to extend the investigation of biological activity to ChEs and MAOs, by testing a limited number of isatin and indole derivatives in in vitro inhibition of human ChEs and MAOs. Compounds were selected according to the following criteria: (a) the most potent inhibitors of Aβ_40_ aggregation within the structural classes collected in Table 1, Table 2, Table 3 and Table 5 (i.e., compounds **9**, including its isomers **6**–**8** for the exploration of putative substitution-activity relationships, **18**, **19**, **23**, **11** and **32**); (b) the most potent inhibitor of tau toxicity (**5**); (c) compounds bearing a basic nitrogen, in order to get an efficient interaction with the catalytic site of ChEs, either at the arylhydrazone moiety (**35**) or Mannich bases at the isatin nitrogen (**18** and **13**).

The data in Table 6 assessed a weak inhibitory potency in AChE inhibition (< 50% at 10 μM), with the valuable exception of benzyloxy isomers **6** and **8** (IC_50_s in the low micromolar range). Disappointingly, none of compounds with basic substituents was found active. As for BChE inhibition, all compounds were ineffective (data not shown).

Some positive results came out from MAOs inhibition tests, with indole derivative **32** acting as a strong inhibitor with submicromolar IC_50_s, although not isoform-selective. Another indole derivative, the quinoline arylhydrazone **35**, showed good inhibition of both isoforms, with a slight preference for MAO A. Isatin phenylhydrazone **5** displayed also good inhibitory potency against MAO A, with 7-fold selectivity over MAO B. *N*-Phenethylisatin **11**, in turn, resulted as the best MAO B selective inhibitor, with IC_50_ in the low micromolar range.

### 2.5. Computational Studies

We carried out computational ligand-based studies, namely pharmacophore modeling and atom-based 3D-QSAR study, on a pool of 93 already published compounds [15,16,17,19] (structures and related Aβ antiaggregation data in Supplementary Materials) for better addressing the design of the new molecules sharing the indole/isatin scaffold. The ligand-based studies were aimed at deriving a comprehensive pharmacophore hypothesis to discern between ‘actives’, i.e., compounds achieving finite IC_50_ values under the threshold 100 μM concentration, and ‘inactives’, i.e. compounds showing no activity or less than 50% inhibition at 100 μM. The pharmacophore was then complemented with a 3D-QSAR study limited to the ‘active’ molecules (IC_50_s < 100 μM), the purpose being to predictively estimate the Aβ inhibition potency of new indole/isatin derivatives and ultimately integrating and improving the physicochemical information content of the pharmacophore for a prospective design.

#### 2.5.1. Top-Scored Pharmacophore Model

**Figure 2:**
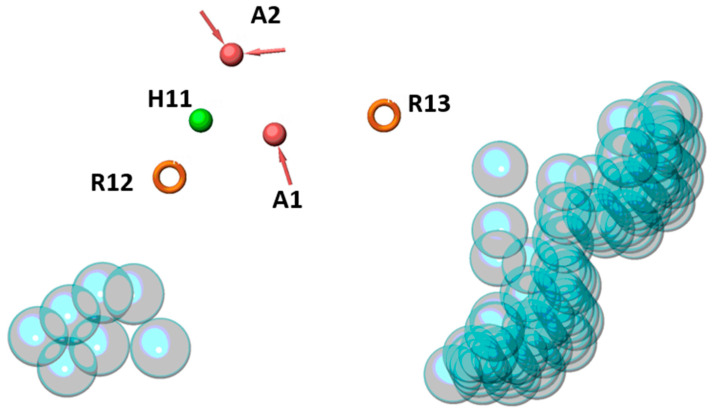
Pharmacophore hypothesis AAHRR. Orange circles, pink and green spheres indicate aromatic rings, hydrogen bonds acceptors and hydrophobic region features, respectively. Excluded volumes are depicted as translucent cyan spheres.

**Figure 3:**
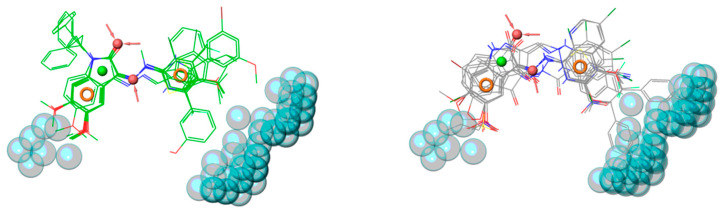
Overlay of actives (in green) and inactives (in gray) shown on the left-hand and right-hand side, respectively.

Five common features were mapped as relevant for the pharmacophore generation: two hydrogen bond acceptors (A), one hydrophobic region (H) and two aromatic rings (R). More specifically, the features of AAHRR pharmacophore were mapped as follows: R12 and H11 refer to isatin, indoline or indandione cores, A2 and A1 refer to the carbonyl of isatin or indandione groups and nitrogen atom of hydrazone moiety, respectively, and R13 refers to phenyl or thiazole rings. Moreover, some excluded volumes were identified which locate sterically disallowed regions (Figure 2). This hypothesis matched 38 out of 54 (70%) ‘actives’ and 39 out of 39 (100%) ‘inactives’, thus having an overall accuracy equal to 82.80% (Figure 3).

**Figure 4:**
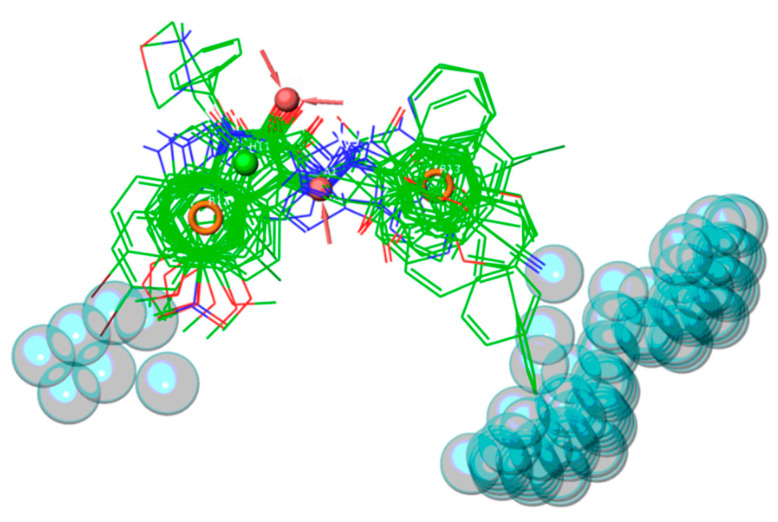
Overlay of the 36 new designed and prepared compounds, based on pharmacophore hypothesis AAHRR. Ligands are rendered as green wireframes.

This pharmacophore hypothesis was used to predict the 36 newly synthesized compounds (Table 1, Table 2, Table 3, Table 4 and Table 5) by setting a minimum number of at least four matched features. Satisfactorily, about 89% of new designed compounds (i.e., 32 out of 36) complied with the AAHRR pharmacophore hypothesis (Figure 4).

#### 2.5.2. 3D-QSAR Model

We derived an atom-based 3D-QSAR model by employing all the compounds achieving finite IC_50_ values below the maximum 100 μM concentration tested. The training set was populated by 54 already reported compounds while the external set by 17 newly synthesized compounds. The first 3D-QSAR run which included all the 54 compounds into the training set did not yield any statistically meaningful model within the first five factors (i.e., PLS components). A careful check of the residuals [38,39] revealed a cluster of nine outliers (residuals > 2*s*): six bearing substituents (both polar and hydrophobic) at the *para* position of the phenyl ring (i.e., **S29**, **S33**, **S49**, **S57** and **S59** in Supplementary Materials) or at position 4 of the thiazole ring (**S69**) of the aryl-hydrazone moiety, and three lipophilic phenyl hydrazones of 3-oxo-3*H*-indole-2-carbaldehyde (**S10**, **S14**, and **S15**). Excluding relevant discrepancies arising from experimental procedures, as on the other hand confirmed by positive controls and re-determination of some representative ‘old’ compounds, the anomalous deviation from the model of these nine molecules may be likely due to the existence of a nonlinear correlation between the inhibition potency and the ligand hydrophobicity, as noticed by correlation with RP-HPLC parameter in our previous work [19]. In the PLS equation of the 3D-QSAR model there are no square neither cross terms which can account for nonlinear effects. Omitting from the PLS regression the nine outliers, the 3D-QSAR model, encompassing 45 molecules (83%) of the whole training set, was able in predicting quite satisfactorily the IC_50_ values of 14 (82%) external test set compounds (r^2^_ext_ = 0.695); three badly predicted compounds were the hydrazone derivatives **30**, **31** and **32**, sharing the 1*H*-indole core, a much less represented scaffold than 1*H*-indole-2,3-dione (i.e., isatin) in the training set.

The final 3D-QSAR model has the following features and statistics: four PLS factors; coefficient of determination r^2^ equal to 0.887 and a standard deviation value to 0.196; leave-one-out coefficient of determination q^2^ equal to 0.596; external coefficient of determination r^2^_ext_ equal to 0.695 and root mean square error (RMSE) value equal to 0.270. Other statistical parameters could be additionally employed to support the prediction capability of the 3D-QSAR model [40]. We preferred validating the model with a true external set of compounds.

**Figure 5:**
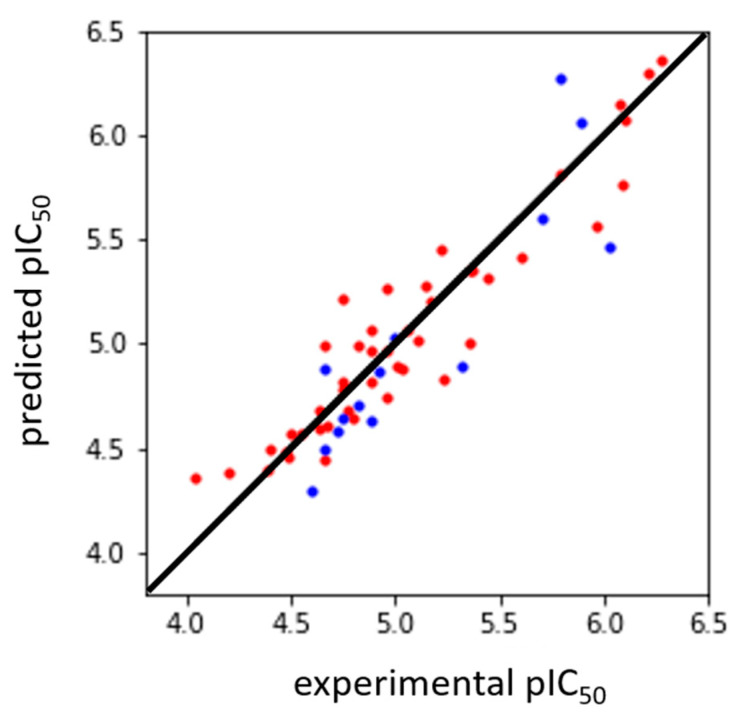
Scatter plot showing experimental vs. predicted pIC_50_ values for training (*n* = 45, red solid circles) and external (*n* = 14, blue solid circles) sets, respectively. Bisector line is depicted in black. The r^2^ and r^2^_ext_ coefficient values were equal to 0.887 and 0.695, respectively.

The grid-based 3D-QSAR model included the following molecular fields: hydrophobic/non-polar, electron-withdrawing and hydrogen bonds, whose relative contributions were equal to 74.3%, 17.7% and 7.5%, respectively. The remaining 0.5% was due to a miscellaneous field [41]. All statistics related to the first four PLS components are summarized in Table 7. Moreover, the correlation between experimental and predicted pIC_50_ values for training and external test sets is shown in Figure 5.

**Figure 6:**
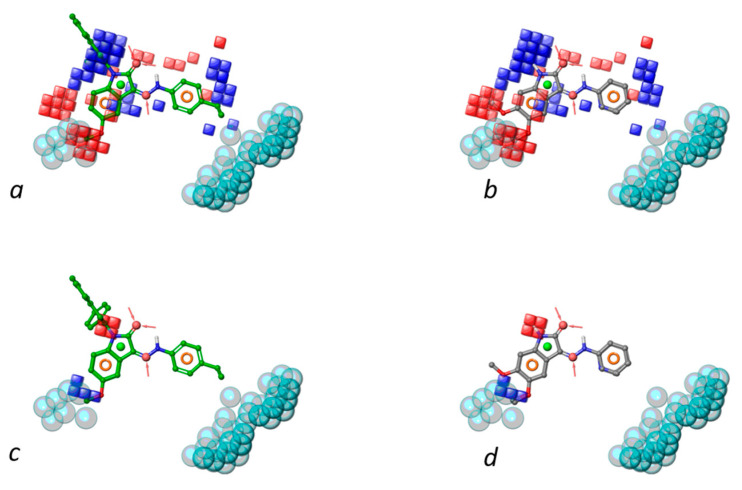

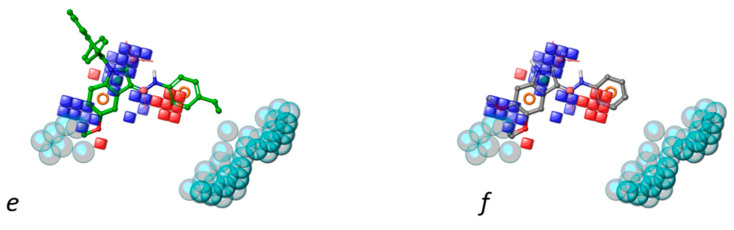
Contour maps rendered as blue and red cubes indicate positive and negative regions for activity. Specifically, panels (**a**,**c**,**e**) show the most active ligands within hydrophobic, HBD and electron withdrawing region, respectively; panels (**b**,**d**,**f**) show the most inactive ligands within hydrophobic, HBD and electron withdrawing regions, respectively. Pharmacophore features and excluded volumes are also reported.

Contour maps of grid-based descriptors are reported in Figure 6 to spot positive or negative molecular regions for the activity of the compounds. Hydrophobic substituents at position 5 and 6 of isatin or indoline cores are detrimental for activity, much more than those at the *ortho* position of the phenyl ring. Furthermore, bulky substituents, such as *iso-*butyl or benzyloxy groups, at the *para* position of phenyl ring cause a drop of the activity and thus impact the excluded volumes of the pharmacophore model. Hydrogen bond donors, such as hydroxyl groups, can enhance the activity at the position 5 and 6 of the isatin core. The carbonyl groups of 3-indolinone or isatin are beneficial for activity, while electron-withdrawing substituents decrease the activity when branching the *ortho* position of the phenyl ring.

## 3. Materials and Methods

### 3.1. General Information

Commercial reagents and solvents were purchased from Sigma-Aldrich (Milan, Italy). Melting points (mp) were determined by the capillary method on a Stuart SMP3 electrothermal apparatus (Bibby Scientific, Milan, Italy). IR spectra were recorded using potassium bromide disks on a Spectrum One FT-IR spectrophotometer (Perkin Elmer, Milan, Italy); only the most significant IR absorption bands are reported. ^1^H-NMR spectra were recorded in DMSO-*d_6_*, CDCl_3_ or acetone-*d_6_* on a Mercury 300 or 500 spectrometer (Varian, Cernusco s. N., Italy). Chemical shifts are expressed in δ (ppm) and the coupling constants *J* in Hz. The following abbreviations were used: s, singlet; d, doublet; t, triplet; qn, quintuplet; ep, eptuplet; dd, double doublet; td, triplet of doublets; m, multiplet; br s, broad singlet. Chromatographic separations were performed on silica gel 63–200 (Merck, Milan, Italy). ESI-MS was performed with an electrospray interface and an ion trap mass spectrometer (1100 Series LC/MSD Trap System, Agilent, Palo Alto, CA, USA). The sample was infused via a KD Scientific syringe pump at a rate of 10 mL/min. The pressure of the nebulizer gas was 15 psi. The drying gas was heated to 350 °C at a flow of 5 L/min. Full-scan mass spectra were recorded in the mass/charge (*m*/*z*) range of 50–800 amu. For some representative compounds, HRMS experiments were performed with a dual electrospray interface (ESI) and a quadrupole time-of-flight mass spectrometer (Q-TOF, Agilent 6530 Series Accurate-Mass Quadrupole Time-of-Flight LC/MS, Agilent Technologies Italia S.p.A., Cernusco sul Naviglio, Italy). Compounds **2** [42], **4** [43]**, 5** [44], **10**–**12** and **14** [19], **30** [45], **33** [15], **44** [23] and **45** [24] have been prepared according to quoted references and their analytical data agreed with those reported in the literature.

### 3.2. Chemistry

#### 3.2.1. Synthesis of (*Z*)-3-Hydrazineylidene-5-nitroindolin-2-one (**3**)

A mixture of 5-nitroisatin (1 mmol, 1 eq) and hydrazine hydrate 55% (2.5 eq) in methanol (1.5 mL) was refluxed for 1 h and then cooled to room temperature, resulting in the precipitation of hydrazone that was filtered, dried and recrystallized from ethanol. Yield: 87%. ^1^H-NMR (DMSO-*d*_6_) δ 6.99 (s, 1H, H-4), 8.10–8.06 (m, 2 H, H-6 and H-7), 10.18 (d, 1H, *J*_v_ = 12.5 Hz, N=NH_2_), 10.72 (d, 1H, *J*_v_ = 12.5 Hz, N=NH_2_), δ 11.30 (br s, 1H, NH indol.). MS (ESI), *m/z* = 204.9 (100%) [M − H]^−^. IR (KBr): 3310, 1624, 1596, 1331 cm^−1^. Mp 217–219 °C.

#### 3.2.2. Synthesis of Aryl-hydrazineylidene indolinones **6**–**9**

Compounds **6**–**9** were synthesized by condensation of *N*-methylisatin or isatin with suitable arylhydrazine hydrochloride [19].

*3-(2-(3-(Benzyloxy)phenyl)hydrazineylidene)indolin-2-one* (**6**) Yield: 60%. ^1^H-NMR (500 MHz, DMSO-*d_6_*) δ 5.12 (s, 2H), 6.67 (dd, 1H, *J* = 8.1, 2.1 Hz), 6.89 (d, 1H, *J* = 7.8 Hz), 6.99–6.94 (m, 1H), 7.03 (t, 1H, *J* = 7.6 Hz), 7.11 (t, 1H, *J* = 2.1 Hz), 7.27–7.19 (m, 2H), 7.31 (t, 1H, *J* = 7.3 Hz), 7.38 (t, 2H, *J* = 7.5 Hz), 7.46 (d, 2H, *J* = 7.3 Hz), 7.54 (d, 1H, *J* = 7.5 Hz), 10.99 (s, 1H), 12.66 (s, 1H). HRMS (ESI) *m*/*z* [M + Na]^+^ calcd for C_21_H_17_N_3_O_2_ 366.1213; found, 366.1195; [M − H]^−^ calcd 342.1248; found, 342.1238. IR (KBr): 3416, 3162, 1557, 1207 cm^−1^. Mp 182–186 °C from EtOH.

*3-(2-(3-(Benzyloxy)phenyl)hydrazineylidene)-1-methylindolin-2-one* (**7**) Yield: 55%. ^1^H-NMR (500 MHz, DMSO-*d_6_*) δ 3.23 (s, 3H), 5.12 (s, 2H), 6.68 (dd, 1H, *J* = 8.1, 2.0 Hz), 6.98 (dd, 1H, *J* = 8.0, 1.3 Hz), 7.10 (t, 2H, *J* = 7.3 Hz), 7.13 (t, 1H, *J* = 2.1 Hz), 7.25 (t, 1H, *J* = 8.1 Hz), 7.31 (dd, 2H, *J* = 11.6, 4.1 Hz), 7.38 (t, 2H, *J* = 7.5 Hz), 7.46 (d, 2H, *J* = 7.4 Hz), 7.61–7.56 (m, 1H), 12.60 (s, 1H). HRMS (ESI) *m*/*z* [M + Na]^+^ calcd for C_22_H_19_N_3_O_2_ 380.1369; found, 380.1374; [M − H]^−^ calcd 356.1405; found, 356.1391. IR (KBr): 3418, 1676, 1567 cm^−1^. Mp 134–137 °C from EtOH.

*3-(2-(4-(Benzyloxy)phenyl)hydrazineylidene)indolin-2-one* (**8**) Yield: 54%. ^1^H-NMR (500 MHz, DMSO-*d_6_*) δ 5.06 (d, 2H, *J* = 5.7 Hz), 6.89 (d, 1H, *J* = 7.8 Hz), 7.02 (dd, 3H, *J* = 12.5, 5.8 Hz), 7.19 (td, 1H, *J* = 7.7, 1.0 Hz), 7.31 (t, 1H, *J* = 7.3 Hz), 7.39–7.33 (m, 4H), 7.43 (d, 2H, *J* = 7.3 Hz), 7.50 (d, 1H, *J* = 7.3 Hz), 10.93 (s, 1H), 12.73 (s, 1H). HRMS (ESI) *m*/*z* [M + Na]^+^ calcd for C_21_H_17_N_3_O_2_ 366.1213; found, 366.1206; [M − H]^−^ calcd 342.1248; found, 342.1239. IR (KBr): 3418, 1680, 1552, 1228, 1174 cm^−1^. Mp 201–206 °C from EtOH.

*3-(2-(4-(Benzyloxy)phenyl)hydrazineylidene)-1-methylindolin-2-one* (**9**) Yield: 50%. ^1^H-NMR (500 MHz, DMSO-*d_6_*) δ 3.23 (s, 3H), 5.07 (s, 2H), 7.02 (d, 2H, *J* = 9.0 Hz), 7.08 (t, 2H, *J* = 7.2 Hz), 7.33–7.25 (m, 2H), 7.37 (dd, 4H, *J* = 8.2, 5.3 Hz), 7.43 (d, 2H, *J* = 7.2 Hz), 7.56–7.50 (m, 1H), 12.67 (s, 1H). HRMS (ESI) *m*/*z* [M + Na]^+^ calcd for C_22_H_19_N_3_O_2_ 380.1369; found, 380.1373; [M − H]^−^ calcd 356.1405; found, 356.1390. IR (KBr): 3435, 1667, 1555, 1218 cm^−1^. Mp 126–128 °C from EtOH.

#### 3.2.3. Alkylation of 5-Methoxyindoline-2,3-diones **13** and **15**

##### *5-Methoxy-1-(morpholinomethyl)indoline-2,3-dione* (**13**)

To a solution of formaldehyde (37% in H_2_O, 4 eq) and morpholine (2 eq) in ethanol (10 mL) was added 5 mL of ethanoic solution of 5-methoxyisatin (1 mmol, 1 eq). The mixture was refluxed for 2 days and after TLC control, the solvent was evaporated, and the residue crystalized. Yield: 61%. ^1^H-NMR (CDCl_3_) δ 2.61 (t, 4H, *J* = 4.7 Hz, CH_2_CH_2_ morpholine), 3.69 (t, 4H, *J* = 4.7 Hz, CH_2_CH_2_ morpholine), 3.81 (s, 3H, OCH_3_), 4.41 (s, 2H, N-CH_2_), 7.02 (d, 1H, *Jo* = 9.1 Hz, H-7), 7.14–7.18 (m, 2H, H-4 and H-6). MS (ESI), *m/z* = 299.0 (100%) [M + Na]^+^. IR (KBr): 2847, 2833, 1738, 1490, 1313 cm^−1^. Mp 107–110 °C from EtOH.

##### *1-(2-(Dimethylamino)ethyl)-5-methoxyindoline-2,3-dione* (**15**)

A solution in DMF (11 mL) of 5-methoxyisatine (2.8 mmol, 1 eq), 2-chloro *N*,*N*-dimethylethylamine hydrochloride (1.3 eq) and potassium carbonate (3 eq) was stirred overnight at room temperature. The mixture was filtered, and the solution was evaporated to afford a red oil that was purified by column chromatography. Yield: 75%. ^1^H-NMR (CDCl_3_) δ 2.29 (s, 6H, N(CH_3_)_2_), 2.57 (t, 2H, *Jv* = 6.9 Hz, CH_2_), 3.83–3.77 (m, 5H, CH_2_ and OCH_3_), 6.87–6.83 (m, 1H), 7.16–7.10 (m, 2H). MS (ESI), *m/z* = 271.10 (100%) [M + Na]^+^. IR (KBr): 2943, 1731, 1484, 1163 cm^−1^. Mp 95–97 °C from CHCl_3_/MeOH 90:10 (*v/v*).

#### 3.2.4. *1-(2-(Dimethylamino)ethyl)-3-(2-(4-isopropylphenyl)hydrazineylidene)-5-methoxyindolin-2-one* (**18**)

Compound **18** was synthesized by condensation of **15** with 4-isopropylphenylhydrazine hydrochloride as previously described [19]. Yield: 35%. ^1^H-NMR (DMSO-*d_6_*) δ 1.14 (d, 6H, *Jv* = 7.1 Hz, CH(CH_3_)_2_), 2.75–2.91 (m, 7H, CH(CH_3_)_2_, N(CH_3_)_2_), 3.30–3.45 (m, 2H, NCH_2_CH_2_N(CH_3_)_2_) 3.79 (s, 3H, OCH_3_), 4.17 (t, 2H, *Jv* = 6.0 Hz, NCH_2_CH_2_N(CH_3_)_2_), 6.90 (dd, 1H, *Jo* = 8.3, *Jm* = 2.3 Hz, H-6), 7.12–7.26 (m, 4H, H-4, H-7, H-3′ and H-5′), 7.39 (d, 2H, *J* = 8.5 Hz, H-2′ and H-6′), 12.60 (s, 1H, NNH). MS (ESI), *m/z* = 379.2 (100%) [M − H]^−^, 403.2 (100%) [M + Na]^+^. IR (KBr): 2959, 1670, 1521, 1485 cm^−1^. Mp 222–224 °C from EtOH/H_2_O.

#### 3.2.5. Synthesis of 5-Hydroxy-isopropylphenyl-hydrazineylidene indolinones **19** and **20**

The debenzylation with BBr_3_ in dichloromethane at −70 °C [16] of compounds **16** and **17** [19] gave the **19** and **20,** respectively.

*1-Benzyl-5-hydroxy-3-(2-(4-isopropylphenyl)hydrazineylidene)indolin-2-one* (**19**). Yield: 20%. ^1^H-NMR (DMSO-*d_6_*) δ 1.19 (d, 6H, *Jv* = 7.0 Hz, CH(CH_3_)_2_), 2.86 (ept, 1H, *Jv* = 7.0 Hz, CH(CH_3_)_2_), 4.94 (s, 2H, CH_2_-Ph), 6.62 (dd, 1H, *Jo* = 8.4, *Jm* = 2.4 Hz, H-6), 6.82 (d, 1H, *Jo* = 8.4 Hz, H-7), 6.98 (d, 1H, *Jm* = 2.4 Hz, H-4), 7.24 (d, 2H, *J* = 8.8 Hz, H-3′ and H-5′), 7.27–7.36 (m, 7H, H-2′, H-6, H-2′′, H-3′′, H-4′′, H-5′′ and H-6′′), 9.21 (s, 1H, OH), 12.71 (s, 1H, NNH). MS (ESI), *m/z* = 383.9 (100%) [M − H]^−^. IR (KBr): 3991, 3032,1655, 1519, 1164 cm^−1^. Mp 188–190 °C from hexane/ethyl acetate 70:30 (*v/v*).

*1-Cyclopropyl-5-hydroxy-3-(2-(4-isopropylphenyl)hydrazono)indolin-2-one* (**20**). Yield: 20%. ^1^H-NMR (DMSO-*d_6_*) δ 0.81–0.86 (m, 2H, CH_2_cyclo-pr.), 0.96–1.03 (m, 2H, CH_2_cyclo-pr.), 1.18 (d, 6H, *Jv* = 7.0 Hz, CH(CH_3_)_2_), 2.73 (qn, 1H, *Jv* = 3.7 Hz, CHcyclopr.), 2.85 (ep, 1H, *Jv* = 7.0 Hz, CH(CH_3_)_2_), 6.72 (dd, 1H, *Jo* = 8.4, *Jm* = 2.6 Hz, H-6), 6.94 (d, 1H, *Jm* = 2.4 Hz, H-4), 6.99 (d, 1H, *Jo* = 8.4 Hz, H-7), 7.22 (d, 2H, *J* = 8.6 Hz, H-3′ and H-5′), 7.31 (d, 2H, *J* = 8.4 Hz, H-2′ and H-6′), 9.21 (s, 1H, OH), 12.74 (s, 1H, NNH). MS (ESI), *m/z* = 333.9 (100%) [M − H]^−^. IR (KBr): 3305, 2920, 1656, 1559, 1390, 1190, 1138 cm^−1^. Mp 222–224 °C from hexane/ethyl acetate 75:25 (*v/v*).

#### 3.2.6. Synthesis of 3-Arylhydrazono-indolin-2-ones **23**–**25**

Compounds **23**–**25** were prepared from azo coupling of aryldiazonium salts **22a**–**c** with indolin-2-one **21**, previously prepared [20]. Aryldiazonium salts were synthesized starting from corresponding amines as described in a previous paper [19].

*3-(2-(Naphthalen-1-yl)hydrazono)indolin-2-one* (**23**). Yield: 20%. ^1^H-NMR (DMSO-*d_6_*) δ 6.97 (d, 1H, *Jo* = 7.7 Hz, H-7), 7.09 (t, 1H, *Jo* = 7.7 Hz, *Jo* = 7.3 Hz, H-5), 7.28 (t, 1H, *Jo* = 7.7 Hz, H-6), 7.54–7.69 (m, 5H, H-3′, H-4´′, H-5′, H-6′and H-7′), 7.82 (d, 1H, *Jo* = 7.3 Hz, H-4), 7.89 (d, 1H, *Jo* = 8.4 Hz, H-2′), 7.98 (d, 1H, *Jo* = 8.1 Hz, H-8′), 11.23 (s, 1H, NH), 13.79 (s, 1H, NNH). MS (ESI), *m/z* = 285.8 (100%) [M − H]^−^. IR (KBr): 3413, 3121, 3049, 1675, 1561, 1196 cm^−1^. Mp 225–230 °C from hexane/ethyl acetate 80:20 (*v/v*).

*3-(2-(Naphthalen-2-yl)hydrazono)indolin-2-one* (**24**). Yield: 30%. ^1^H-NMR (DMSO-*d_6_*) δ 6.92 (d, 1H, *Jo* = 7.7 Hz, H-7), 7.06 (t, 1H, *Jo* = 7.7 and 7.3 Hz, H-5), 7.25 (td, 1H, *Jm* = 1.1 Hz, *Jo* = 7.7 Hz, H-6), 7.36 (t, 1H, *Jo* = 8.1 and 7.0 Hz, H-6′), 7.41 (t, 1H, *Jo* = 8.1 and 7.0 Hz, H-7′), 7.61 (d, 1H, *Jo* = 7.3 Hz, H-4), 7.71 (dd, 1H, *Jm* = 1.8 Hz, *Jo* = 8.8 Hz, H-3′), 7.83–7.86 (m, 3H, H-1′, H-5′ and H-8′), 7.93 (d, 1H, *Jo* = 8.8 Hz, H-4′), 11.05 (s, 1H, NH), 12.95 (s, 1H, NNH). MS (ESI), *m/z* = 285.8 (100%) [M − H]^−^. IR (KBr): 3159, 1676, 1555, 1223, 1190 cm^−1^. Mp 245–248 °C from hexane/ethyl acetate 70:30 (*v/v*).

*3-(2-(Quinolin-8-yl)hydrazineylidene)indolin-2-one* (**25**). Yield: 21%.^1^H-NMR (DMSO-*d_6_*) δ 6.92 (d, 1H, *Jo* = 7.5 Hz, H-7), 7.06 (t, 1H, *Jo* = 7.7 Hz, H-5), 7.26 (t, 1H, *Jo* = 7.7 Hz, H-6), 7.58–7.67 (m, 4H, H-3′, H-4′, H-6′and H-4), 7.93 (dd, 1H, *Jo* = 7.0, *Jm* = 2.2 Hz, H-5′), 8.39 (dd, 1H, *Jo* = 8.4, *Jm* = 1.6 Hz, H-2′), 8.91–8.93 (m, 1H, H-7′), 11.07 (s, 1H, NH), 13.91 (s, 1H, NNH). MS (ESI), *m/z* = 311.0 (100%) [M + Na]^+^. IR (KBr): 3447, 3152, 1682, 1523, 1198 cm^−1^. Mp > 250 °C dec. from CH_2_Cl_2_/MeOH 96:4 (*v/v*).

#### 3.2.7. *N′-(5-Methoxy-2-oxoindolin-3-ylidene)benzhydrazide* (**26**)

The compound was synthesized by condensation of 5-methoxyisatin and benzhydrazide as previously described [19]. Yield: 37%. ^1^H-NMR (DMSO-*d_6_*) δ 3.77 (s, 3H, 5-OCH_3_), 6.87 (d, 1H, *J* = 8.6 Hz, H-7), 6.96 (dd, 1H, *Jo* = 8.6, *Jm* = 2.6 Hz, H-6), 7.13 (d, 1H, *Jm* = 2.6 Hz, H-4), 7.59 (dd, 2H, *Jo* = 8.4, *Jm* = 1.5 Hz, H-5′ and H3′), 7.63–7.61 (m, 1H, H-4′), 7.88 (dd, 2H, *Jo* = 8.4, *Jm* = 1.5 Hz, H-2′ and H-6′), 11.20 (br s, 1H, NH), 13.90 (br s, 1H, NNHCO). ESI-MS *m*/*z* = 318.0 (100%) [M + Na]^+^, 293.8 (100%) [M − H]^−^. IR (KBr): 3254, 1704, 1691, 1675, 1486 cm ^−1^. Mp > 250 °C from CH_2_Cl_2_/MeOH 96:4 (*v*/*v*).

#### 3.2.8. *2-(4-Hydroxybenzylidene)indolin-3-one* (**27**)

Compound **27** was prepared following literature procedures [46]. Yield: 25%. ^1^H-NMR (DMSO-*d_6_*) δ 6.60 (s, 1H, =CH), 6.85 (d, 2H, *Jo* = 8.7 Hz, H-3′and H-5′), 6.87 (t, 1H, *Jo* = 7.6 Hz, H-5), 7.11 (d, 1H, *Jo* = 8.3 Hz*,* H-7), 7.48 (td, 1H, *Jo* = 8.8, *Jm* = 1.1 Hz, H-6), 7.54 (d, 1H, *Jo* = 7.6 Hz, H-4), 7.68 (d, 2H, *Jo* = 8.7 Hz, H-2′and H-6′), 9.54 (s, 1H, NH), 9.98 (br s, 1H, OH). MS (ESI), *m/z* = 235.9 (100%) [M − H]^−^. IR (KBr): 3311, 1672, 1591, 1509, 1488, 1243 cm^−1^. Mp > 250 °C.

#### 3.2.9. Synthesis of Hydrazonomethyl-indoles **28**, **29**, **31**, **32**, **34**–**36**

The synthesis of hydrazonomethyl-indoles was performed as previously described [19] by the condensation of corresponding indole carbaldehyde and arylhydrazine. Then the desired compounds were purified by crystallization or column chromatography.

*3-((2-(4-Isopropylphenyl)hydrazono)methyl)-1H-indole* (**28**). Yield: 30%. ^1^H-NMR (DMSO-*d_6_*) δ 1.16 (d, 6H, *J* = 7.0 Hz, CH(CH_3_)_2_), 2.80 (ep, 1H, *J* = 7.0 Hz, CH(CH_3_)_2_), 6.96–7.40 (m, 6H, H-6, H-7, H-2′, H-3′, H-5′ and H-6′), 7.59 (s, 1H, H-2), 8.08 (s, 1H, H-8), 8.21–8.24 (m, 1H, H-4), 8.64–8.65 (m, 1H, H-5), 11.31 (s, 1H, NH), 12.79 (s, 1H, NNH). MS (ESI), *m/z* = 275.9 (100%) [M − H]^−^. IR (KBr): 3436, 2841, 1644, 1489, 1428, 1223, 1146, 751 cm^−1^. Mp 200–205 °C from EtOH.

*4-(2-((5-Methoxy-1H-indol-3-yl)methylene)hydrazineyl)benzonitrile* (**29**). Yield: 30%. ^1^H-NMR (DMSO-*d_6_*) δ 3.82 (s, 3H, OCH_3_), 6.84 (dd, 1H, *Jm* = 2.6, *Jo* = 8.8 Hz, H-6), 7.06 (d, 2H, *J* = 8.6 Hz, H-2′ and H-6′), 7.31 (d, 1H, *Jo* = 8.8 Hz, H-7), 7.59 (d, 2H, *J* = 8.6 Hz, H-3′ and H-5′), 7.69 (s, 1H, H-2), 7.71 (d, 1H, *Jm* = 2.6 Hz, H-4), 8.17 (s, 1H, CH=N), 10.55 (s, 1H, NH), 11.33 (s, 1H, NNH). MS (ESI), *m/z* = 288.8 (100%) [M − H]^−^. IR (KBr): 3290, 2220, 1603, 1532, 1290, 1258 cm^−1^. Mp 227–229 °C from EtOH.

*3-((2-(3-Chlorophenyl)hydrazineylidene)methyl)-5-methoxy-1H-indole* (**31**). Yield: 30%. ^1^H-NMR (DMSO-*d_6_*) δ 3.84 (s, 3H, OCH_3_), 6.66 (dd, 1H, *Jm* = 2.1, *Jo* = 8.3 Hz, H-4′), 6.82 (dd, 1H, *Jm* = 2.6, *Jo* = 8.8 Hz, H-6), 6.86 (dd, 1H, *Jm* = 2.1, *Jo* = 8.3 Hz, H-6′), 7.11 (t, 1H, *Jm* = 2.1 Hz, H-2′), 7.18 (t, 1H, *Jo* = 8.3, H-5′), 7.30 (d, 1H, *Jo* = 8.8 Hz, H-7), 7.61 (br s, 1H, H-2), 7.75 (d, 1H, *Jm* = 2.6 Hz, H-4), 8.09 (s, 1H, CH=N), 10.08 (s, 1H, NH), 11.23 (s, 1H, NNH). MS (ESI), *m/z* = 297.8 (100%) [M − H]^−^, 299.8 (33%) [M − H]^−^+2. IR (KBr): 3430, 2922, 1614, 1596, 1484, 1210 cm^−1^. Mp 83–85 °C from ethyl acetate/hexane 50:50 (*v/v*).

*3-((2-(4-Chlorophenyl)hydrazono)methyl)-1H-indole* (**32**). Yield: 30%. ^1^H-NMR (acetone-*d_6_*) δ 7.15 (d, 2H, *J* = 9.1 Hz, H-2′ and H-6′), 7.23 (d, 2H, *J* = 9.1 Hz, H-3′ and H-5′), 7.13–7.25 (m, 2H, H-6 and H-7), 7.43–7.47 (m, 1H, H-4), 7.59 (s, 1H, H-2), 8.17 (s, 1H, H-8), 8.36–8.42 (m, 1H, H-5), 9.13 (s, 1H, NH), 10.47 (s, 1H, NNH). MS (ESI), *m/z* = 267.9 (100%) [M − H]^−^, 269.8 (39%) [M − H]^−^ + 2. IR (KBr): 3412, 3302, 1599, 1497, 1433, 1074, 747 cm^−1^. Anal. calcd for C_15_H_12_N_3_Cl: C, 66.79; H, 4.48; N, 15.58. Found: C, 66.45; H, 5.14; N, 12.11. Mp 117–122 °C from ethyl acetate/hexane 50:50 (*v/v*).

*5-Methoxy-3-((2-(pyridin-2-yl)hydrazineylidene)methyl)-1H-indole* (**34**). Yield: 50%. ^1^H-NMR (DMSO-*d_6_*) δ 3.82 (s, 3H, OCH_3_), 6.64–6.67 (m, 1H, H-4′), 6.82 (dd, 1H, *Jo* = 8.8, *Jm* = 2.5 Hz, H-6), 7.15 (d, 1H, *Jo* = 8.2 Hz, H-6′), 7.30 (d, 1H, *Jo* = 8.8 Hz, H-7), 7.60 (br s, 1H, H-2), 7.63–7.66 (m, 1H, H-5′), 7.74 (d, 1H, *Jm* = 2.5 Hz, H-4), 8.04–8.08 (m, 1H, H-3′), 8.21 (s, 1H, CH=N), 10.41 (s, 1H, NH), 11.24 (s, 1H, NNH). MS (ESI), *m/z* = 264.9 (100%) [M − H]^−^, 289.0 (100%) [M + Na]^+^. IR (KBr): 3439, 3184, 2998, 1598, 1444 cm^−1^. Mp 185–187 °C from EtOH.

*2-(2-((5-Methoxy-1H-indol-3-yl)methylene)hydrazineyl)quinoline* (**35**). Yield: 50%. ^1^H-NMR (DMSO-*d_6_*) δ 3.87 (s, 3H, OCH_3_), 6.84 (dd, 1H, *Jo* = 8.8, *Jm* = 2.6 Hz, H-6), 7.20–7.24 (m, 1H), 7.32 (d, 1H, *Jo* = 8.8 Hz, H-7), 7.50–7.73 (m, 5H), 7.80 (d, 1H, *Jm* = 2.2 Hz, H-4), 8.17 (d, 1H, *Jo* = 9.2 Hz), 8.27 (s, 1H, CH=N), 10.96 (s, 1H, NH), 11.28 (s, 1H, NNH). MS (ESI), *m/z* = 314.8 (100%) [M − H]^−^, 317.0 (100%) [M + Na]^+^, 339.0 (42%) [M+H]^+^. IR (KBr): 3432, 2948, 1607, 1571, 1430 cm^−1^. Mp 233–235 °C from EtOH.

*7-Chloro-4-(2-((5-methoxy-1H-indol-3-yl)methylene)hydrazineyl)quinoline* (**36**). Yield: 44%. ^1^H-NMR (DMSO-*d_6_*) δ 3.86 (s, 3H, OCH_3_), 6.87 (d, 1H, *Jo* = 8.4 Hz, H-6), 7.24–7.26 (m, 1H), 7.36 (d, 1H, *Jo* = 8.4 Hz, H-7), 7.54 (d, 1H, *Jo* = 8.4 Hz), 7.65–7.87 (m, 3H), 8.41–8.44 (m, 3H), 8.64 (br s, 1H, CH=N), 11.51 (br s, 1H, NNH). MS (ESI), *m/z* = 348.9 (100%) [M − H]^−^, 373.0 (100%) [M + Na]^+^, 351.0 (16%) [M+H]^+^. IR (KBr): 3351, 3231, 1615, 1578, 1425 cm^−1^. Mp >255 °C from EtOH.

#### 3.2.10. *3-(2-(4-Isopropylphenyl)hydrazineylidene)isoindolin-1-one* (**38**)

The 3-iminoisoindolinone **37** previously prepared [47] (1 mmol, 1 eq) was dissolved in 5 mL of methanol, then 4-isopropylphenylhydrazine (2.4 eq) was added at room temperature. After stirring overnight, the mixture was filtered and the precipitate was purified by crystallization. Yield: 60%. ^1^H-NMR (DMSO-*d_6_*) δ 1.16 (d, 6H, *J* = 7.0 Hz, CH(CH_3_)_2_), 2.80 (ep, 1H, *J* = 7.0 Hz, CH(CH_3_)_2_), 7.03 (d, 2H, *J* = 8.6 Hz, H-2′ and H-6′), 7.13 (d, 2H, *J* = 8.6 Hz, H-3′ and H-5′), 7.53 (td, 1H, *Jo* = 7.4, *Jm* = 1.1 Hz, H-5), 7.68 (td, 1H, *Jo* = 7.4, *Jm* = 1.1 Hz, H-6), 7.75 (dd, 1H, *Jo* = 7.4, *Jm* = 0.8 Hz, H-4), 7.83 (dd, 1H, *Jo* = 7.4, *Jm* = 0.8 Hz, H-7), 9.31 (s, 1H, NH), 10.72 (s, 1H, NNH). ESI-MS *m*/*z* = 277.9 (100%) [M–NH]^−^. IR (KBr): 3351, 2964, 1712, 1612, 1127 cm^−1^. Mp 186–187 °C from CHCl_3_/hexane.

#### 3.2.11. Synthesis of 5-Bromo-3-chloro-2-((2-phenylhydrazineylidene)methyl)-1*H*-indoles **40** and **41**

Compound **39** (0.3 mmol, 1 eq), previously synthesized by conversion of acyclic dicarboxylic to aldehyde under Vilsmeier condition [21], was solubilized in ethanol (1 M). Catalytic amount of acetic acid and suitable arylhydrazine (1.2 eq) were added to the solution and the mixture was stirred for 4 h at 0 °C. Then the solvent was evaporated, and the crude product was crystalized or purified by column chromatography.

*5-Bromo-3-chloro-2-((2-phenylhydrazineylidene)methyl)-1H-indole* (**40**). Yield: 40%. ^1^H-NMR (CDCl_3_) δ 6.93 (t, 1H, Jo = 7.4 Hz, H-4′), 7.12 (d, 2H, *Jo* = 8.8, H-2′ and H-6′), 7.22 (d, 1H, *Jo* = 8.8 Hz, H-7), 7.27–7.35 (m, 3H), 7.72 (br s, 1H, CH=N), 7.82 (s, 1H, H-4), 7.86 (s, 1H, NNH), 8.71 (s, 1H, NH). MS (ESI), *m/z* = 346.0 (54%) [M − H]^−^, 347.8 (100%) [M − H]^−^ +2, 349.7 (25%) [M − H]^−^ +4. IR (KBr): 3415, 1630, 1460 cm^−1^. Mp 165–170 °C from CHCl_3_/hexane.

*5-Bromo-3-chloro-2-((2-(4-isopropylphenyl)hydrazineylidene)methyl)-1H-indole* (**41**). Yield: 20%. ^1^H-NMR (DMSO-*d_6_*) δ 1.16 (d, 6H, *Jv* = 6.9 Hz, CH(CH_3_)_2_), 2.80 (ep, 1H, *Jv* = 6.9 Hz, CH(CH_3_)_2_), 7,07–7.12 (m, 4H, H-2′, H-3′, H-4′, H-5′ and H-6′), 7.28 (dd, 1H, *Jo = 8.8, Jm* = 1.8 Hz, H-6), 7.36 (d, 1H, *Jo* = 8.8 Hz, H-7), 7.55 (d, 1H, *Jm* = 1.8 Hz, H-4), 7.89 (s, 1H, CH=N), 10.64 (s, 1H, NH), 11.66 (s, 1H, NNH). MS (ESI), *m/z* = 387.8 (73%) [M − H]^−^, 389.7 (100%) [M − H]^−^ +2, 391.7 (26%) [M − H]^−^ +4. IR (KBr): 3382, 2956, 1537, 1515, 1254 cm^−1^. Mp 140–143 °C from hexane/ethyl acetate 90:10 (*v/v*).

#### 3.2.12. 2-(2,4-Dihydroxyquinolin-3-yl)-3*H*-indol-3-one (**43**)

The dihydroxyquinolinedione **42** [22] (0.5 mmol, 1 eq) was mixed with indoxyl acetate (1 eq) in neat condition and heated to 120 °C. After 15 min the mixture was cooled and dissolved with diethyl ether. The organic solvent was decanted and evaporated obtaining the desired compound. Yield: 45%. ^1^H-NMR (DMSO-*d_6_*) δ 6.90 (t, 1H, *Jo* = 7.4 Hz), 7.04–7.12 (m, 4H), 7.38 (d, 1H, *Jo* = 8.3 Hz), 7.58 (t, 1H, *Jo* = 7.7 Hz), 7.70 (d, 1H, *Jo* = 7.7 Hz), 10.97 (s, 1H, OH), 11.05 (s, 1H, OH). MS (ESI), *m/z* = 289.8 (100%) [M − H]^−^. IR (KBr): 3385, 1713, 1673, 1614 cm^−1^. Mp 90–92 °C.

### 3.3. Biological Assays

All assays were performed in 96-well plates (Greiner Bio-One, Kremsmünster, Austria) using an Infinite M1000 Pro plate reader (Tecan, Cernusco s.N., Italy). Inhibition data were obtained as mean ± SD (for single concentration points) or mean ± SEM from three independent experiments (for IC_50_s), using Prism (GraphPad Prism version 5.00 for Windows, GraphPad Software, San Diego, CA, USA).

#### 3.3.1. Inhibition of Aβ_40_ Self-Aggregation

Assay of aggregation of Aβ_40_ (EzBiolab, Carmel, IN, USA) was performed as described [25], using 2% of 1,1,1,3,3,3-hexafluoro-2-propanol as aggregation enhancer and spectrofluorimetric detection of thioflavin T fluorescence as the probe of amyloid aggregation.

#### 3.3.2. Inhibition of Cholinesterases

Inhibition of human recombinant AChE (2770 U/mg) or BChE from human serum (50 U/mg; both from Sigma Aldrich, St. Louis, MO, USA), was determined as described [48], by applying the Ellman’s spectrophotometric method to a 96-well plate procedure.

#### 3.3.3. Inhibition of Monoamine Oxidases

Inhibition of human recombinant monoamine oxidases A (250 U/mg) and B (59 U/mg; microsomes from baculovirus infected insect cells; Sigma Aldrich) was determined as already described [48], measuring the fluorescence of 4-hydroxyquinoline produced by the oxidative deamination of substrate kynuramine.

### 3.4. In Vitro Cell-Based Assays

Compounds for bacterial media were purchased from CondaLab (Madrid, Spain). M9 minimal medium; for 100 mL: 10 mL salts 10× (0.68 g Na_2_HPO_4_, 0.30 g KH_2_PO_4_, 0.05 g NaCl, 0.10 g NH_4_Cl), 0.2 mL 1M MgSO_4_, 0.2 mL 50 mM CaCl_2_, 2.5 mL 20% glucose and 87.1 mL H_2_O.

#### 3.4.1. Tau Aggregation Inhibition Assay in Bacterial Cells

*Escherichia coli* competent cells BL21 (DE3) were transformed with pTARA containing the RNA-polymerase gen of T7 phage (T7RP) under the control of the promoter PBAD and pRKT42 vector encoding four repeats of tau protein in two inserts. Because of the addition of the initiation codon ATG in front of gene, the over-expressed protein contains an additional methionine residue at its N terminus. Tau anti-aggregating activities of the target compounds were assessed in *E. coli* cells, as previously described [27,28,29]. M9 minimal medium (10 mL) containing 0.5% of glucose, 100 μg/mL of ampicillin and 12.5 μg/mL of chloramphenicol was inoculated with a colony of BL21 (DE3) cells bearing the plasmids. To fresh M9 minimal medium containing 0.5% of glucose, 50 μg/mL of ampicillin, 12.5 μg/mL of chloramphenicol, and 250 µM of ThS, the volume of overnight culture necessary to get a 1:500 dilution was added. The cultures were grown at 37 °C and 250 rpm overnight until cell density reached OD_600_ = 0.6. A volume of 980 μL of the cultures was transferred into 1.5 mL Eppendorf tubes that contained 10 μL of a solution of the target compound in DMSO and 10 μL of arabinose at 25%, leading to a final inhibitor concentration of 10 μM. The resulting cultures were grown at 37 °C and 1400 rpm with a Thermomixer (Eppendorf, Hamburg, Germany) overnight. The same amount of DMSO without the target compound was added to the sample as a negative control (maximal amount of tau), whereas non-induced samples (in the absence of arabinose) were prepared as positive controls (absence of tau), and to assess the potential intrinsic toxicity of the target compounds. In addition, the absorbance at 600 nm of these samples was assessed to confirm the correct bacterial growth, discarding potential intrinsic toxicity of compounds. To determine the IC_50_ values, the same protocol was followed but only modifying the initial concentration of compounds. In order to use different final concentrations of compound, we performed a dilutions banc from a stock solution of 100 mM in MilliQ H_2_O.

#### 3.4.2. Thioflavin S Steady-State Fluorescence

ThS fluorescence and absorbance were tracked using a DTX 800 plate reader Multimode Detector equipped with a Multimode Analysis Software (Beckman-Coulter, Indianapolis, IN, USA). Filters of 430/35 and 485/20 nm were used for the excitation and emission wavelengths, respectively. 535/25 nm filters were also used for the absorbance determination. It should be stressed that ThS fluorescence normalization was carried out considering as 100% the ThS fluorescence of the bacterial cells expressing tau in the absence of compounds and 0% the ThS fluorescence of the bacterial cells non-expressing tau.

### 3.5. Computational Studies

For each compound a suitable three-dimensional structure was built by employing MacroModel [49] for energy minimization based on OPLS_2005 force field and by setting a maximum of 100 bioactive conformers as set in ConfGen [50]. Ligprep [51] was employed for determining possible protonation states at physiological pH. The pharmacophore and atom-based 3D-QSAR models were then derived by using Phase [41] after entering the prepared structures along with their respective biological activity values. The ligands were thus designated as actives or inactives based on the IC_50_ threshold equal to 100 μM. Flexible ligands alignment was automatically performed by Phase. The maximum and minimum number of features was set to 7 and 4, respectively, with a tolerance sphere of 2 Å, provided that at least 50% of actives must match. Excluded volumes were also accounted to better discern inactives for which the clash value was set to 1 and the minimum distance between active surface and excluded volume was set to 1 Å. In doing that, 13 different pharmacophore hypotheses were generated sorted on the values of the Phase Hypo Score, which is a linear combination of different contributes related with site, volume, vector and selectivity scores [52,53].

3D-QSAR model was based on partial least squares (PLS) regression statistics on grid descriptors (that are HBA, HBD, hydrophobic interactions and electron withdrawing effects) sampled on a lattice with a spacing of 1 Å. The number of optimal PLS factors was equal to 4 given that the increase of q^2^ was not higher than 5% by adding a further component. For the sake of clarity, only compounds with experimentally measured IC_50_ values were included in the analysis.

## 4. Conclusions

Building on our previous results, we progressed the decoration of the isatin scaffold to prepare a large number of derivatives displaying antiamyloidogenic activity over a wide range of potency. Five out of the 36 new compounds displayed IC_50_ values in the low to submicromolar range, being *N*-substituted isatins **9** (IC_50_ = 0.95 μM), **18** (1.3 μM) and **19** (1.6 μM) the most potent inhibitors of Aβ aggregation. This feature was in full agreement with our previous observations [19] of the enhancing effect brought by *N*-substitution at the isatin nitrogen, as herein confirmed by comparing the pairs **6**–**7** and **8**–**9**. The screening of selected compounds in tau overexpressing cells allowed to disclose an interesting inhibitory activity against tau aggregation, although no apparent correlation was found between this activity and in vitro inhibition of Aβ aggregation. Among the tested compounds, isatin 3-phenylhydrazone **5** scored the best activity (4.6 μM). Taking into account our previous observations assessing the low intrinsic toxicity of isatin scaffold in different cellular assays [17,18,19], we may conclude that this class of molecules has the potential for further development as antiamyloidogenic agents with safe cytotoxic profile.

In this paper, we also expanded the exploration of SAR to indole derivatives, most of them bearing the arylhydrazone moiety (compounds **28**–**36**) and resulting with few exceptions in good inhibitors of Aβ aggregation. Other structural variations based on molecular simplification or shift of hydrazone substituent were not favorable for activity.

In the perspective of discovering new multitarget agents for AD, we selected some candidates for their evaluation as inhibitors of AChE and MAOs, two target enzymes involved in neurodegenerative diseases. Satisfactorily, several isatin and indole derivatives displayed interesting MAO inhibition, overall selective (compounds **9**, **11**, **13**) towards the B isoform. Unsurprisingly, isatin hydrazone **5** resulted a potent MAO A inhibitor, since the MAO activity has been reported for different isatin derivatives [35,36,37]. Conversely, we found indole derivative **32** as the most potent, although nonselective, MAO inhibitor with IC_50_ values of 0.34 and 0.23 μM for MAO A and MAO B, respectively. Even considering its good potency in inhibition of Aβ aggregation (8.4 μM), the multitarget profile of **32** as dual MAO/Aβ aggregation inhibitor deserves further insights, mainly aiming to improve A/B selectivity.

The data obtained from in vitro tests of inhibition of Aβ aggregation prompted us to derive a pharmacophore model for Aβ antiaggregating activity. The molecular shape, lipophilicity of substituents, and the substitution at the isatin nitrogen appeared to be the main determinants for antiamyloidogenic activity. The combined approach based on pharmacophore and 3D-QSAR models allowed us to furnish more confident predictions thus allowing to better address the rational design of new active and more promising compounds.

## Figures and Tables

**Table 1:**
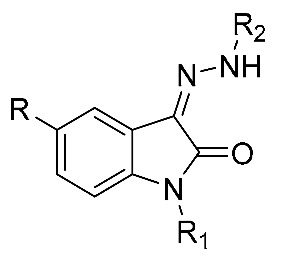
Inhibition data of new isatin derivatives **18**–**20** and **23**–**26**.

Comp.	R	R_1_	R_2_	Aβ_40_ Aggregation Inhibition	
				% at 100 μM	IC_50_ (μM)
**18**	5-OCH_3_	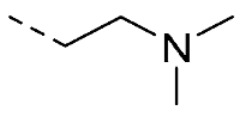	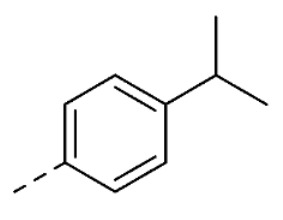		1.3 ± 0.1
**19**	5-OH	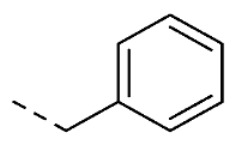			1.6 ± 0.3
**20**	5-OH	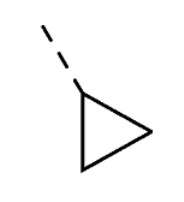		26 ± 3	
**23**	H	H	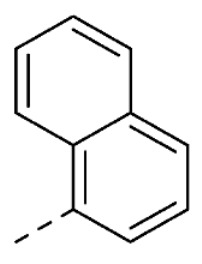		4.8 ± 0.5
**24**	H	H	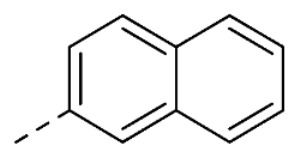		10 ± 2
**25**	H	H	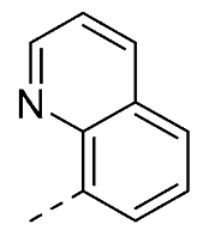	63 ± 5	
**26**	5-OCH_3_	H	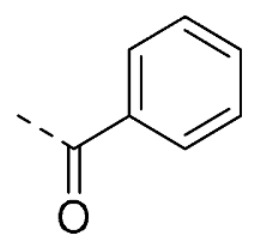	12 ± 5	
**1**					0.43 ^a^
**Quercetin**					0.82 ^a^

^a^ Data taken from [17]. Values are mean ± SEM (*n* = 3).

**Table 2:**
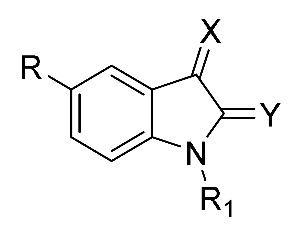
Inhibition data of new isatin derivatives **2**, **3**, **10**–**14**, **27** and **44**.

Code	R	R_1_	X	Y	Aβ_40_ Aggregation Inhibition	
					% at 100 μM	IC_50_ (μM)
**2**	H	H	N-NH_2_	O	17 ± 2	
**3**	5-NO_2_	H	N-NH_2_	O	11 ± 3	
**10**	5-OCH_3_	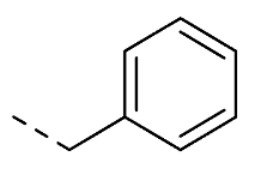	O	O	52 ± 1	
**11**	5-OCH_3_	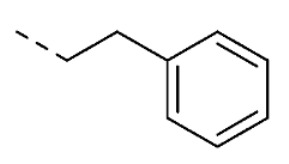	O	O		15 ± 1
**12**	5-OCH_3_	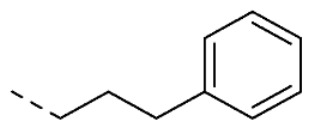	O	O		19 ± 4
**13**	5-OCH_3_	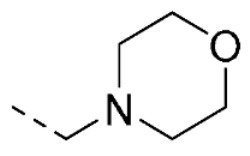	O	O	23 ± 5	
**14**	5-OCH_3_	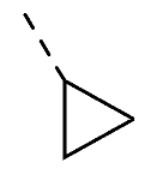	O	O	38 ± 5	
**27**	H	H	O	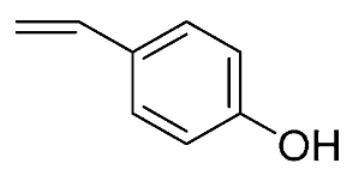	69 ± 1	
**44**	H	H	O	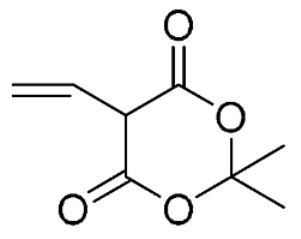	44 ± 1	

**Table 3:**
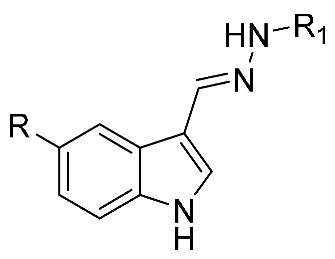
Inhibition data of new indole-3-carbaldehyde hydrazones **28**–**36** and **45**.

Comp.	R	R_1_	Aβ_40_ Aggregation Inhibition	
			% at 100 μM	IC_50_ (μM)
**28**	H	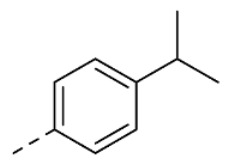		18 ± 2
**29**	5-OCH_3_	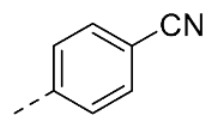	56 ± 1	
**30**	H	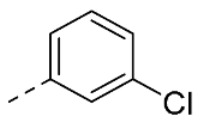		13 ± 2
**31**	5-OCH_3_			9.2 ± 0.7
**32**	H	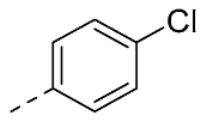		8.4 ± 0.6
**33**	5-OCH_3_			22 ± 2
**34**	5-OCH_3_	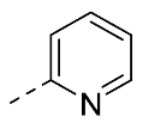	20 ± 2	
**35**	5-OCH_3_	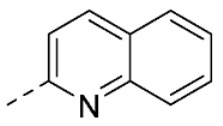		12 ± 2
**36**	5-OCH_3_	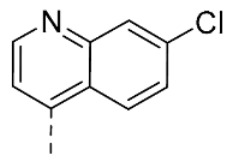		13 ± 1
**45**	5-OCH_3_	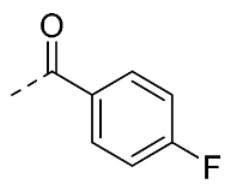	0	

**Table 4:** Inhibition data of new derivatives **38**, **40**, **41** and **43**.

Comp.	Structure	Aβ_40_ Aggregation Inhibition	
		% at 100 μM	IC_50_ (μM)
**38**	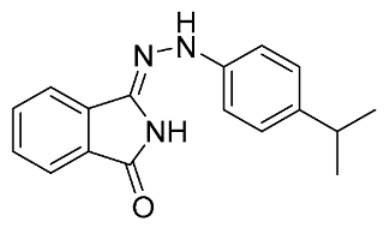	49 ± 5	
**40**	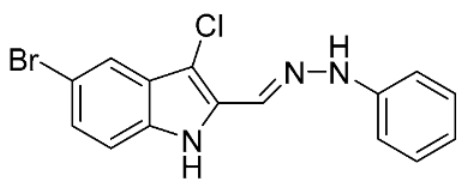		25 ± 3
**41**	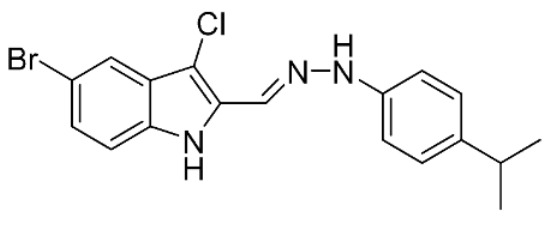	63 ± 6	
**43**	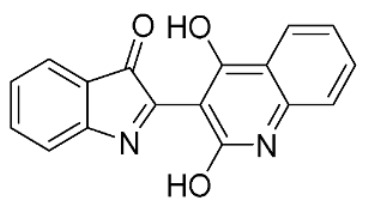	17 ± 3	

**Table 5:** Inhibition of β-amyloid and tau aggregation.

Code	Structure	Aβ_40_ Inhibition		Tau Inhibition ^a^	
		% at 100 μM	IC_50_ (μM)	% at 10 μM	IC_50_ (μM)
**S22 ** ^**b**^	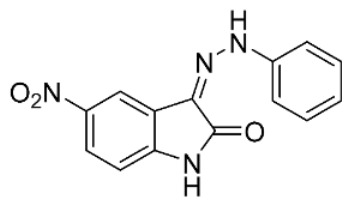	-^c^		15 ± 3	
**S23 ** ^**b**^	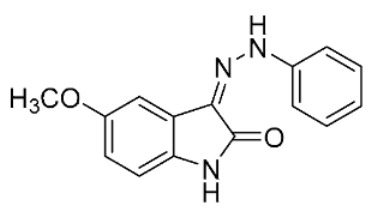	52			7.7 ± 1.0
**S34 ** ^**b**^	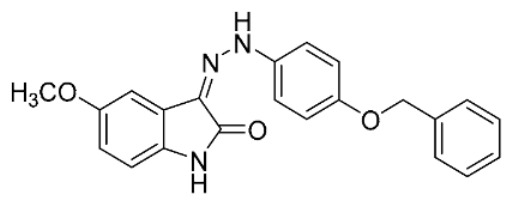	20		16 ± 2	
**S35 ** ^**b**^	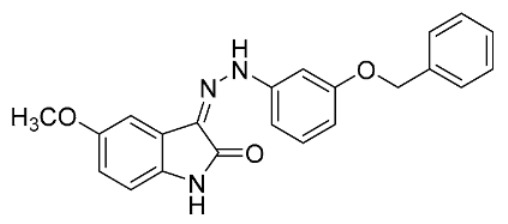	44			10 ± 2
**4**	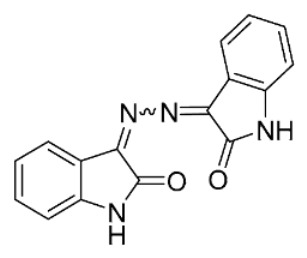	31 ± 3		17 ± 2	
**5**	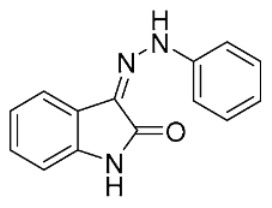		22 ± 2		4.6 ± 0.7
**6**	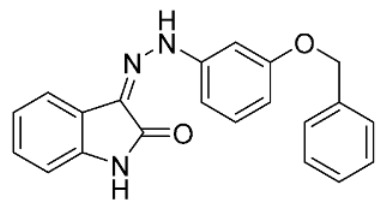	45 ± 6		14 ± 2	
**7**	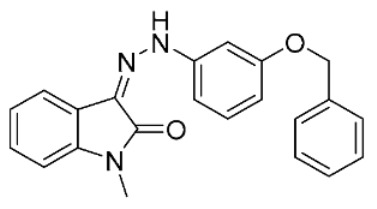		2.0 ± 0.3	19 ± 2	
**8**	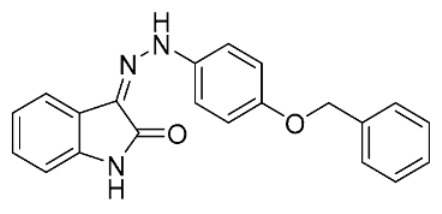	69 ± 3		35 ± 2	
**9**	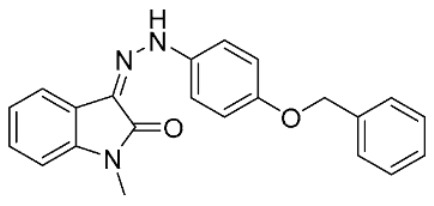		0.95 ± 0.04	20 ± 2	

^a^ From cell-based assay. ^b^ Compounds and their data of β-amyloid aggregation are taken from ref. [16] and presented in Supplementary Materials. Values are mean ± SEM (*n* = 3) in in vitro assay. ^c^ No inhibition at 100 μM concentration.

**Table 6:** Inhibition data of acetylcholinesterase and monoamine oxidases.

Comp.	*h*AChE ^a^		*h*MAO A ^a^		*h*MAO B ^a^	
	% Inhib. at 10 μM	IC_50_ (μM)	% Inhib. at 10 μM	IC_50_ (μM)	% Inhib. at 10 μM	IC_50_ (μM)
**5**	37 ± 1			0.52 ± 0.04		3.6 ± 0.7
**6**		4.2 ± 0.8	23 ± 5		22 ± 4	
**7**	48 ± 3		29 ± 3		41 ± 1	
**8**		3.9 ± 0.5	8 ± 3		36 ± 4	
**9**	48 ± 2		15 ± 3			4.0 ± 0.4
**18**	23 ± 1		31 ± 4		47 ± 2	
**19**	31 ± 1		18 ± 4		46 ± 5	
**23**	40 ± 3			7.1 ± 0.3		5.3 ± 0.3
**11**	47 ± 5		20 ± 4			2.7 ± 0.3
**13**	26 ± 1		7 ± 2			4.4 ± 0.1
**32**	41 ± 5			0.34 ± 0.01		0.23 ± 0.03
**35**	38 ± 2			3.8 ± 0.3		6.5 ± 0.6
**Galantamine**		0.72 ± 0.05	-	-	-	-
**Safinamide**	-	-	18 ± 3			0.031 ± 0.001

^a^ Human recombinant enzymes. Values are mean ± SEM (*n* = 3).

**Table 7:** Atom based 3D-QSAR statistics.

# Factors	SD	r^2^	q^2^	RMSE	r^2^_ext_
1	0.383	0.533	0.320	0.290	0.651
2	0.283	0.751	0.439	0.250	0.754
3	0.231	0.838	0.563	0.260	0.720
4	0.196	0.887	0.596	0.270	0.695

## Supplementary Materials

The following are available online, Chemical structures, SMILES codes, and inhibition values of 93 previously published compounds constituting the training set.
