# Supplementary material for: Pharmacophore Modeling and 3D-QSAR Study of Indole and Isatin Derivatives as Antiamyloidogenic Agents Targeting Alzheimer’s Disease
Source: Molecules. 2020 Dec 7;25(23):5773. doi: 10.3390/molecules25235773 (PMC7731220; doi:10.3390/molecules25235773)
Supplement: Supplementary file 1 [file molecules-25-05773-s001.pdf]

Code	Structure	SMILES	% inhib. @ 100 µM	IC$_{50}$ (µM)	ref.	core structure
**S1**	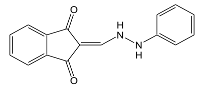	O=C(C1=CC=CC=C1C/2=O)C2=C/NNC3=CC=CC=C3	49±2.0		15	
**S2**	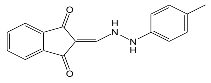	O=C(C1=CC=CC=C1C/2=O)C2=C/NNC3=CC=C(C)C=C3	44±4.0		15	
**S3**	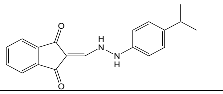	O=C(C1=CC=CC=C1C/2=O)C2=C/NNC3=CC=C(C(C)C)C=C3		**18**	15	
**S4**	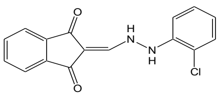	O=C(C1=CC=CC=C1C/2=O)C2=C/NNC3=CC=CC=C3Cl		**32**	15	Indandione
**S5**	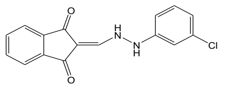	O=C(C1=CC=CC=C1C/2=O)C2=C/NNC3=CC=CC(Cl)=C3		**18**	15	
**S6**	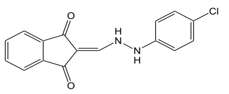	O=C(C1=CC=CC=C1C/2=O)C2=C/NNC3=CC=C(Cl)C=C3		**23**	15	
**S7**	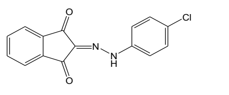	O=C(C1=CC=CC=C1C/2=O)C2=N\NC3=CC=C(Cl)C=C3	38±5.3		15	
**S8**	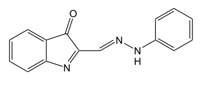	O=C1C(/C=N/NC2=CC=CC=C2)=NC3=CC=CC=C31		**8,8**	15	
**S9**	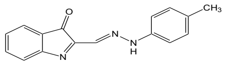	O=C1C(/C=N/NC2=CC=C(C)C=C2)=NC3=CC=CC=C31		**3,6**	15	
**S10**	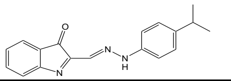	O=C1C(/C=N/NC2=CC=C(C(C)C)C=C2)=NC3=CC=CC=C31		**1,4**	15	
**S11**	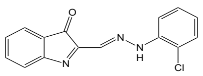	O=C1C(/C=N/NC2=CC=CC=C2Cl)=NC3=CC=CC=C31		**13**	15	
**S12**	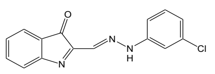	O=C1C(/C=N/NC2=CC=CC(Cl)=C2)=NC3=CC=CC=C31		**2,5**	15	
**S13**	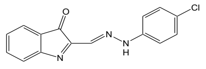	O=C1C(/C=N/NC2=CC=C(Cl)C=C2)=NC3=CC=CC=C31	33±1.0		15	
**S14**	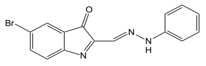	O=C1C(/C=N/NC2=CC=CC=C2)=NC3=CC=C(Br)C=C31		**35**	15	3-indolinone
**S15**	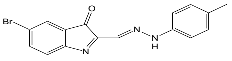	O=C1C(/C=N/NC2=CC=C(C)C=C2)=NC3=CC=C(Br)C=C31		**51**	15	
**S16**	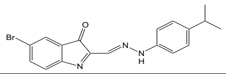	O=C1C(/C=N/NC2=CC=C(C(C)C)C=C2)=NC3=CC=C(Br)C=C31		**23**	15	
**S17**	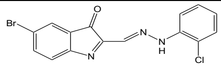	O=C1C(/C=N/NC2=CC=CC=C2Cl)=NC3=CC=C(Br)C=C31		**7,8**	15	
**S18**	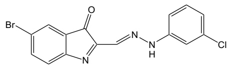	O=C1C(/C=N/NC2=CC=CC(Cl)=C2)=NC3=CC=C(Br)C=C31		**4,4**	15	
**S19**	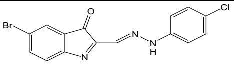	O=C1C(/C=N/NC2=CC=C(Cl)C=C2)=NC3=CC=C(Br)C=C31	46±7.0		15	
**S20**	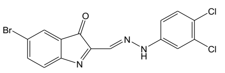	O=C1C(/C=N/NC2=CC=C(Cl)C(Cl)=C2)=NC3=CC=C(Br)C=C31	23±2.3		15	
**S21**	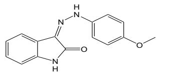	O=C1NC2=CC=CC=C2/C1=N/NC3=CC=C(OC)C=C3		**13**	16	
**S22**	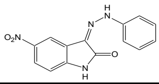	O=C1NC2=CC=C([N+]([O-])=O)C=C2/C1=N/NC3=CC=CC=C3	-2±7		16	

**S23**	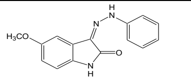	O=C1NC2=CC=C(OC)C=C2/C1=N/NC3=CC=CC=C3	52±8		16
**S24**	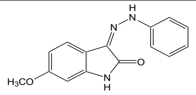	O=C1NC2=CC(OC)=CC=C2/C1=N/NC3=CC=CC=C3		**5,8**	16
**S25**	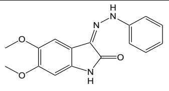	O=C1NC2=CC(OC)=C(OC)C=C2/C1=N/NC3=CC=CC=C3		**63**	16
**S26**	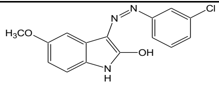	O=C1NC2=CC=C(OC)C=C2/C1=N/NC3=CC(Cl)=CC=C3	*72±1*		16
**S27**	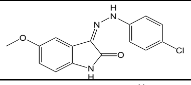	O=C1NC2=CC=C(OC)C=C2/C1=N/NC3=CC=C(Cl)C=C3	43±2		16
**S28**	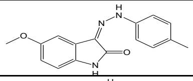	O=C1NC2=CC=C(OC)C=C2/C1=N/NC3=CC=C(C)C=C3	29±4		16
**S29**	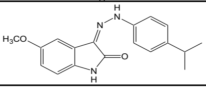	O=C1NC2=CC=C(OC)C=C2/C1=N/NC3=CC=C(C(C)C)C=C3		**0,43**	16
**S30**	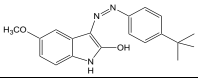	O=C1NC2=CC=C(OC)C=C2/C1=N/NC3=CC=C(C(C)(C)C)C=C3	*66±4*	**15**	16
**S31**	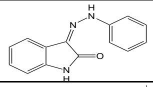	O=C1NC2=CC=CC=C2/C1=N/NC3=CC=CC=C3	(I) 36±2.2 (II) 61±0.7		16
**S32**	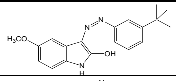	O=C1NC2=CC=C(OC)C=C2/C1=N/NC3=CC(C(C)(C)C)=CC=C3	56±2		16
**S33**	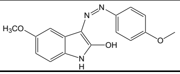	O=C1NC2=CC=C(OC)C=C2/C1=N/NC3=CC=C(OC)C=C3		**3,7**	16
**S34**	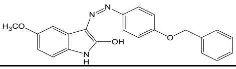	O=C1NC2=CC=C(OC)C=C2/C1=N/NC3=CC=C(OCC4=CC=CC=C4)C=C3	20±1		16
**S35**	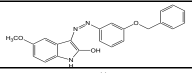	O=C1NC2=CC=C(OC)C=C2/C1=N/NC3=CC=CC(OCC4=CC=CC=C4)=C3	44±4 (I)		16
**S36**	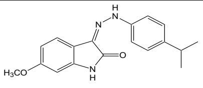	O=C1NC2=CC(OC)=CC=C2/C1=N/NC3=CC=C(C(C)C)C=C3		**7,1**	16
**S37**	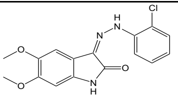	O=C1NC2=CC(OC)=C(OC)C=C2/C1=N/NC3=C(Cl)C=CC=C3	*70±3*		16
**S38**	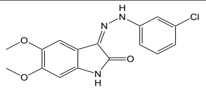	O=C1NC2=CC(OC)=C(OC)C=C2/C1=N/NC3=CC(Cl)=CC=C3		**17**	16
**S39**	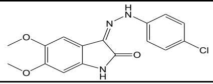	O=C1NC2=CC(OC)=C(OC)C=C2/C1=N/NC3=CC=C(Cl)C=C3		**33**	16
**S40**	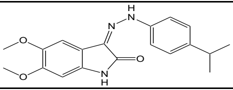	O=C1NC2=CC(OC)=C(OC)C=C2/C1=N/NC3=CC=C(C(C)C)C=C3		**4,4**	16
**S41**	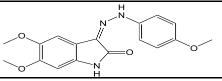	O=C1NC2=CC(OC)=C(OC)C=C2/C1=N/NC3=CC=C(OC)C=C3		**22**	16
**S42**	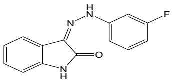	O=C1NC2=CC=CC=C2/C1=N/NC3=CC(F)=CC=C3	52±0.9		16
**S43**	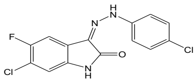	O=C1NC2=CC(Cl)=C(F)C=C2/C1=N/NC3=CC=C(Cl)C=C3	28±3.7		16
**S44**	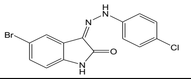	O=C1NC2=CC=C(Br)C=C2/C1=N/NC3=CC=C(Cl)C=C3	63±4		16

**S45**	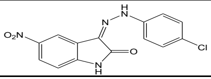	O=C1NC2=CC=C([N+]([O-])=O)C=C2/C1=N/NC3=CC=C(Cl)C=C3	12±2		16	
**S46**	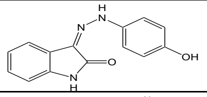	O=C1NC2=CC=CC=C2/C1=N/NC3=CC=C(O)C=C3		**9,3**	16	
**S47**	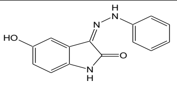	O=C1NC2=CC=C(O)C=C2/C1=N/NC3=CC=CC=C3	62±3		16	
**S48**	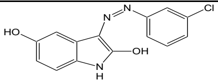	O=C1NC2=CC=C(O)C=C2/C1=N/NC3=CC(Cl)=CC=C3		**18**	16	
**S49**	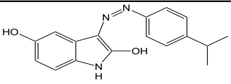	O=C1NC2=CC=C(O)C=C2/C1=N/NC3=CC=C(C(C)C)C=C3		**21**	16	
**S50**	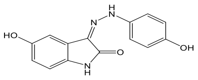	O=C1NC2=CC=C(O)C=C2/C1=N/NC3=CC=C(O)C=C3	61±2		16	
**S51**	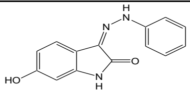	O=C1NC2=CC(O)=CC=C2/C1=N/NC3=CC=CC=C3		**22**	16	
**S52**	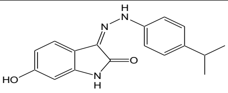	O=C1NC2=CC(O)=CC=C2/C1=N/NC3=CC=C(C(C)C)C=C3		**6**	16	
**S53**	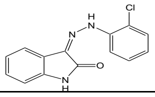	O=C1NC2=CC=CC=C2/C1=N/NC3=C(Cl)C=CC=C3	44±5		16	
**S54**	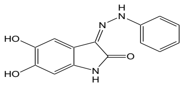	O=C1NC2=CC(O)=C(O)C=C2/C1=N/NC3=CC=CC=C3		**4,3**	16	
**S55**	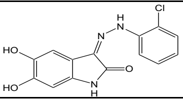	O=C1NC2=CC(O)=C(O)C=C2/C1=N/NC3=C(Cl)C=CC=C3		**11**	16	
**S56**	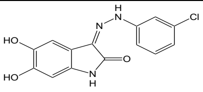	O=C1NC2=CC(O)=C(O)C=C2/C1=N/NC3=CC(Cl)=CC=C3		**1,1**	16,18	
**S57**	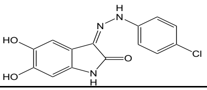	O=C1NC2=CC(O)=C(O)C=C2/C1=N/NC3=CC=C(Cl)C=C3		**17**	16	
**S58**	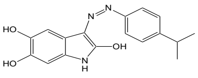	O=C1NC2=CC(O)=C(O)C=C2/C1=N/NC3=CC=C(C(C)C)C=C3		**1,6**	16	
**S59**	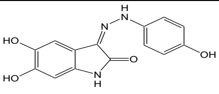	O=C1NC2=CC(O)=C(O)C=C2/C1=N/NC3=CC=C(O)C=C3		**24**	16	
**S60**	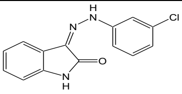	O=C1NC2=CC=CC=C2/C1=N/NC3=CC(Cl)=CC=C3	39±5		16	Isatin 3-hydrazones
**S61**	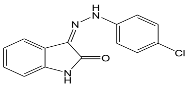	O=C1NC2=CC=CC=C2/C1=N/NC3=CC=C(Cl)C=C3	24±4.9		16	
**S62**	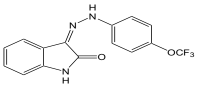	O=C1NC2=CC=CC=C2/C1=N/NC3=CC=C(OC(F)(F)F)C=C3	57±3		16	
**S63**	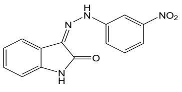	O=C1NC2=CC=CC=C2/C1=N/NC3=CC([N+]([O-])=O)=CC=C3	29±1.2		16	
**S64**	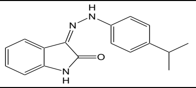	O=C1NC2=CC=CC=C2/C1=N/NC3=CC=C(C(C)C)C=C3	64±4		16	

**S65**	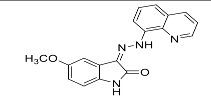	O=C1NC2=CC=C(OC)C=C2/C1=N/NC3=C(N=CC=C4)C4=CC=C3	*58 ± 4 (SD)*		19
**S66**	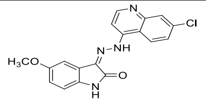	O=C1NC2=CC=C(OC)C=C2/C1=N/NC3=C(C=CC(Cl)=C4)C4=NC=C3	*14 ± 12,5 (SD)*		19
**S67**	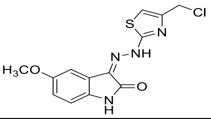	O=C1NC2=CC=C(OC)C=C2/C1=N/NC3=NC(CCl)=CS3		**13**	19
**S68**	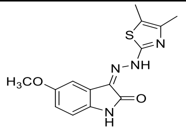	O=C1NC2=CC=C(OC)C=C2/C1=N/NC3=NC(C)=C(C)S3	*43 ± 1.0 (SD)*		19
**S69**	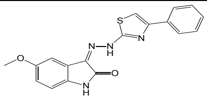	O=C1NC2=CC=C(OC)C=C2/C1=N/NC3=NC(C4=CC=CC=C4)=CS3		**1,21**	19
**S70**	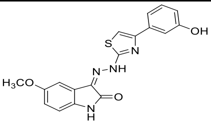	O=C1NC2=CC=C(OC)C=C2/C1=N/NC3=NC(C4=CC(O)=CC=C4)=CS3		**16**	19
**S71**	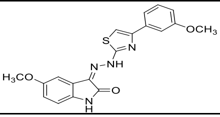	O=C1NC2=CC=C(OC)C=C2/C1=N/NC3=NC(C4=CC(OC)=CC=C4)=CS3		**34**	19
**S72**	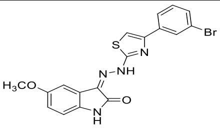	O=C1NC2=CC=C(OC)C=C2/C1=N/NC3=NC(C4=CC(Br)=CC=C4)=CS3		**40**	19
**S73**	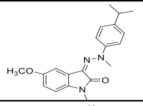	O=C1N(C)C2=CC=C(OC)C=C2/C1=N/N(C)C3=CC=C(C(C)C)C=C3		**11**	19
**S74**	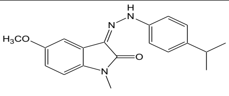	O=C1N(C)C2=CC=C(OC)C=C2/C1=N/NC3=CC=C(C(C)C)C=C3		**0,81**	19
**S75**	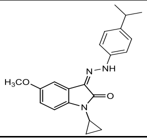	O=C1N(C2CC2)C3=CC=C(OC)C=C3/C1=N/NC4=CC=C(C(C)C)C=C4		**0,8**	19
**S76**	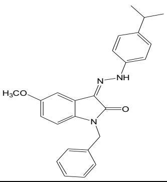	O=C1N(CC2=CC=CC=C2)C3=CC=C(OC)C=C3/C1=N/NC4=CC=C(C(C)C)C=C4		**0,53**	19
**S77**	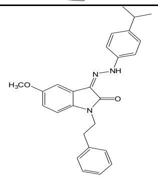	O=C1N(CCC2=CC=CC=C2)C3=CC=C(OC)C=C3/C1=N/NC4=CC=C(C(C)C)C=C4		**0,61**	19
**S78**	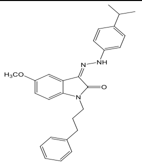	O=C1N(CCCC2=CC=CC=C2)C3=CC=C(OC)C=C3/C1=N/NC4=CC=C(C(C)C)C=C4		**0,83**	19

**S79**	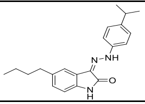	O=C1NC2=CC=C(CCCC)C=C2/C1=N/NC3=CC=C(C(C)C)C=C3	*55±0.9 (SD)*		19	
**S80**	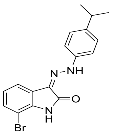	O=C1NC2=C(Br)C=CC=C2/C1=N/NC3=CC=C(C(C)C)C=C3	*1±9.3 (SD)*		19	
**S81**	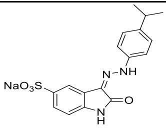	O=C1NC2=CC=C(S([O-])(=O)=O)C=C2/C1=N/NC3=CC=C(C(C)C)C=C3	*35±4.1 (SD)*		19	
**S82**	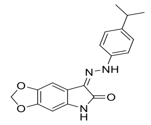	O=C1NC2=CC3=C(OCO3)C=C2/C1=N/NC4=CC=C(C(C)C)C=C4	*70±2.9 (SD)*		19	
**S83**	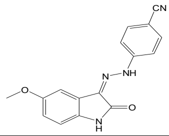	O=C1NC2=CC=C(OC)C=C2/C1=N/NC3=CC=C(C#N)C=C3	*16±3.0 (SD)*		19	
**S84**	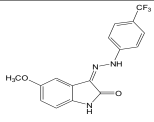	O=C1NC2=CC=C(OC)C=C2/C1=N/NC3=CC=C(C(F)(F)F)C=C3	*57±9.0 (SD)*		19	
**S85**	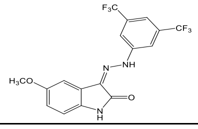	O=C1NC2=CC=C(OC)C=C2/C1=N/NC3=CC(C(F)(F)F)=CC(C(F)(F)F)=C3		**9,9**	19	
**S86**	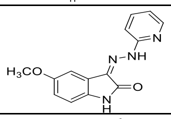	O=C1NC2=CC=C(OC)C=C2/C1=N/NC3=NC=CC=C3		**90**	19	
**S87**	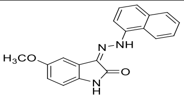	O=C1NC2=CC=C(OC)C=C2/C1=N/NC3=C(C=CC=C4)C4=CC=C3		**28**	19	
**S88**	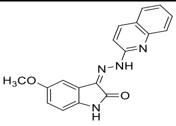	O=C1NC2=CC=C(OC)C=C2/C1=N/NC3=NC(C=CC=C4)=C4C=C3	*67% ± 3,8 (SD)*		19	
**S89**	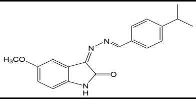	O=C1NC2=CC=C(OC)C=C2/C1=N/N=C/C3=CC=C(C(C)C)C=C3		**6,7**	19	
**S90**	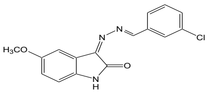	O=C1NC2=CC=C(OC)C=C2/C1=N/N=C/C3=CC(Cl)=CC=C3		**11**	19	
**S91**	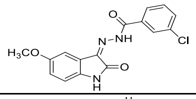	O=C1NC2=CC=C(OC)C=C2/C1=N/NC(C3=CC(Cl)=CC=C3)=O	*31±2.9 (SD)*		19	Other
**S92**	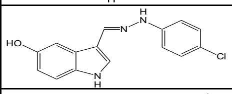	OC1=CC=C(NC=C2/C=N/NC3=CC=C(Cl)C=C3)C2=C1		**21**	15	
**S93**	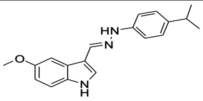	COC1=CC=C(NC=C2/C=N/NC3=CC=C(C(C)C)C=C3)C2=C1		**41**	19	
